# Genome-wide study of hair colour in UK Biobank explains most of the SNP heritability

**DOI:** 10.1038/s41467-018-07691-z

**Published:** 2018-12-10

**Authors:** Michael D. Morgan, Erola Pairo-Castineira, Konrad Rawlik, Oriol Canela-Xandri, Jonathan Rees, David Sims, Albert Tenesa, Ian J. Jackson

**Affiliations:** 1 MRC WIMM Centre for Computational Biology, MRC Weatherall Institute of Molecular Medicine, University of Oxford, John Radcliffe Hospital, Headington, Oxford, OX3 9DS UK; 2 MRC Human Genetics Unit, Institute of Genetics and Molecular Medicine, University of Edinburgh, Crewe Road, Edinburgh, EH4 2XU UK; 3 Roslin Institute, University of Edinburgh, Easter Bush, Midlothian, EH25 9RG UK; 4 Dermatology, University of Edinburgh, Lauriston Building, Edinburgh, EH3 9HA UK; 5 Present Address: Wellcome Trust Sanger Institute, Wellcome Genome Campus, Hinxton, Cambridge, CB10 1SA UK

## Abstract

Natural hair colour within European populations is a complex genetic trait. Previous work has established that *MC1R* variants are the principal genetic cause of red hair colour, but with variable penetrance. Here, we have extensively mapped the genes responsible for hair colour in the white, British ancestry, participants in UK Biobank. *MC1R* only explains 73% of the SNP heritability for red hair in UK Biobank, and in fact most individuals with two MC1R variants have blonde or light brown hair. We identify other genes contributing to red hair, the combined effect of which accounts for ~90% of the SNP heritability. Blonde hair is associated with over 200 genetic variants and we find a continuum from black through dark and light brown to blonde and account for 73% of the SNP heritability of blonde hair. Many of the associated genes are involved in hair growth or texture, emphasising the cellular connections between keratinocytes and melanocytes in the determination of hair colour.

## Abstract (web summary)

Natural hair colour in Europeans is a complex genetic trait. Here, the authors carry out a genome-wide association study using UK BioBank data, suggesting that in combination with pigmentation genes, variants with roles in hair texture and growth can affect hair colouration or our perception of it.

## Introduction

Natural hair colour within European populations is strikingly variable and is a complex genetic trait that is impacted relatively little by known, non-genetic, factors^1^. Furthermore, hair colour is largely determined by only a few well-characterised cell types: the melanocytes where the melanin pigment is made, the keratinocytes of the hair to which the pigment is transferred and fibroblasts of the dermal papilla, which signal to and regulate the melanocytes. It is thus an excellent model system to explore genetic and cellular interactions in development and homoeostasis. Hair colour variation is partially correlated with skin and eye colour variation, reflecting differences in cellular interaction in different tissues^2,3^.

Several studies have examined the genetic basis of hair colour variation. Red hair is well established as being associated with coding variation in the *MC1R* gene^4,5^. Less well known is the observation that most of these variants are only partially penetrant, and some of them have very low penetrance indeed^6^. Other genetic factors must be interacting with *MC1R* to modify the penetrance of these variants. MC1R is a G-protein-coupled receptor, expressed on the surface of skin and hair melanocytes. Binding of the MC1R cognate ligand, α-melanocyte stimulating hormone (α-MSH), induces a melanogenic cascade resulting in the production of dark eumelanin. This is packaged into vesicles, termed melanosomes, for transport to epidermal keratinocytes where it provides protection against ultraviolet radiation. The cellular trafficking of melanosomes to keratinocytes in the hair follicle additionally gives colour to the growing hair. Loss of MC1R signalling in many vertebrate species results in the inability of the melanocytes to produce eumelanin that instead default to synthesising phaeomelanin, a red or yellow pigment. MC1R has a second ligand, an inverse agonist, agouti signalling protein (ASIP)^7^. Overexpression of ASIP, in mice for example, leads to synthesis of only yellow phaeomelanin, even in the presence of a functional MC1R and α-MSH^8^. Previous studies have defined a role for *ASIP* in red hair in humans, but its molecular basis is largely unknown^9,10^.

Until recently, genome-wide association studies (GWAS) identified only a small number of loci associated with blonde hair, compared to black and brown^11–14^. Each of these studies identified between 4 and 8 genes, with a total of 11 genes associated with hair colour differences. Most of these genes have been previously described as causing coat colour variation in mice (*MC1R*, *ASIP*, *OCA2*, *SLC45A2*, *KITLG*, *TYR*, *TYRP1*, *EDNRB*), zebrafish (*SLC24A5*) and humans (*TPCN2*, *IRF4*). However, during preparation of this manuscript a GWAS was reported identifying over 100 loci contributing to hair colour^15^. Hysi et al.^15^ analysed a subset of participants in a very large population health cohort of British individuals, UK Biobank, in addition to a similar number of individuals from 23andMe, totalling 290,891. This report, however, mostly considered hair colour as a single ordered variable including red hair. This has the potential to mask associations due to differences in genetic architecture for separate categories of hair colour.

We report here the analysis of the majority of UK Biobank, a total of almost 350,000 subjects. By performing genome-wide analyses across hair colours, we have discovered novel variation in and around *MC1R* that contributes to red hair. Furthermore, we identify eight additional variants that explain most of the SNP heritability of red hair, including variants at *ASIP*, where an eQTL shows epistatic interactions with the poorly penetrant *MC1R* variants. Additional epistatic interactions are seen between *MC1R* and the *HERC2/OCA2* locus and with *PKHD1*. Furthermore, we identify more than 200 genetic variants independently associated with multiple hair colours on the spectrum of blond to black. Notably, we find that many of the associated genes seem not to be involved in melanocyte biology per se, but are rather involved in hair growth or texture. This highlights the importance of the melanocyte–keratinocyte interactions in the determination of hair pigmentation and the impact of hair shape on colour perception.

## Results and Discussion

### UK Biobank

Participants in UK Biobank responded to the question “what is your natural hair colour” with one of six possible answers. We used only self-reported, white British individuals, confirmed by genotype^16^. In addition, of the individuals who were third-degree relatives (first cousins) or closer, identified by genotyping^16^, only one of any related group was analysed. This left 343,234 participants with hair colours shown in Table 1. The white non-British individuals and the relatives removed from the primary analysis were subsequently analysed to validate the genetic risk scores that we derive.

**Table 1:** Number and percentages of hair colours in the UK Biobank cohort, by gender

	Male	Female	Total
Red	6033 (3.8%)	9698 (5.2%)	15,731 (4.6%)
Blonde	15,838 (10.0%)	23,559 (12.7%)	39,397 (11.5%)
Light brown	62,908 (40.0%)	78,506 (42.5%)	141,414 (41.1%)
Dark brown	59,150 (37.2%)	68,830 (37.2%)	127,980 (37.2%)
Black	11,784 (7.4%)	2737 (1.5%)	14,526 (4.2%)
Other/NA	3238 (2.0%)	1598 (0.9%)	4836 (1.4%)
Total	158,951	184,928	343,884

Generally the frequencies of different hair colours are comparable to other populations with considerable Northern European ancestry, such as the QIMR cohort from Australia^15^, but as expected the prevalence of red hair is higher and black hair is lower than in Southern European cohorts. Within UK Biobank a higher proportion of females report red or blonde hair than males and a much lower proportion of females report black hair. Whilst there may be some self-reporting bias, we and others have previously shown using colorimetry that females on average have hair that is both more red and lighter^3,17^.

Genotypes for more than 800,000 SNPs and indels were directly assayed by UK Biobank using custom Affymetrix arrays, and an additional ~40 M variants imputed using the Haplotype Reference Consortium panel^16^.

### Red hair colour and MC1R

**Fig. 1:**
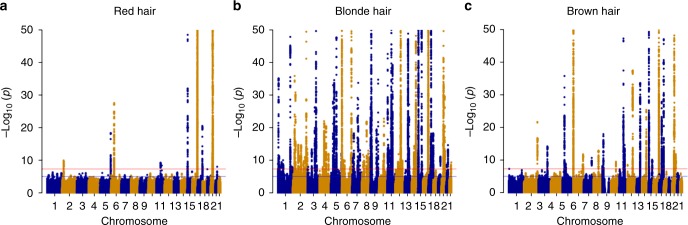
Manhattan plots of GWAS data. Data plotted for **a** red hair vs. black plus brown hair, **b** blonde hair vs. black plus brown hair, **c** brown hair vs. black hair. Points are truncated at –log10(*p*) = 50 for clarity

We performed a GWAS comparing individuals with red hair to a combined group of black- and brown-haired individuals. Accounting for genetic structure within the UK Biobank by inclusion of the first 15 genetic principal components adequately controlled the genomic inflation in our analysis (*λ*_GC_ 1.024). The strongest association with red hair is located around the *MC1R* gene on human chromosome 16 (Fig. 1a, Supplementary Table 1, Supplementary Figure 1a), which fits with the expectation that this locus is the principal genetic factor determining red hair colour. We find that the strongest signal of association in the region of *MC1R* (rs34357723; OR 9.59, *p* < 2.25×10^−308^) does not originate from any observed amino acid changes, but is an SNP located some 97 kb from the 5′ end of *MC1R* and remains significant even after adjusting for all coding variants in MC1R. As we know that multiple *MC1R* alleles affect red hair colour, we performed stepwise conditional association testing and identified 31 additional association signals in this region at genome-wide levels of statistical significance (*p* ≤ 5×10^−8^), altering the odds of having red hair compared to brown and black hair (Supplementary Table 1). Only ten association signals can be directly attributed to amino acid changes, nonsense or frameshift mutations within the *MC1R* coding region. Included in these are two missense variants rs368507952 (R306H) and rs200000734 (R213W) not previously associated with red hair colour.

In addition to these 10 coding variants, we find 21 associations beyond the *MC1R* coding region at distances up a megabase both 3′ and 5′. These distant associations have been observed in other studies^2,12^. Although these variants potentially could affect long-range regulatory elements of *MC1R*, it is likely that they are synthetic associations caused by low linkage disequilibrium (LD) between the associated SNPs and multiple coding variants.

We asked how many cases of red hair can be accounted for by MC1R coding variation. We included rs3212379, located only 120 bp 5′ of the transcription start site of *MC1R*, as a candidate transcriptional regulatory variant. Including this variant, the two newly associated missense variants described above and 13 previously described coding variants we find that the proportion of red-haired individuals with two *MC1R* alleles is 92%, whilst only 6.3% carry a single allele. The cases of red hair with only one or no variants (similar to that seen in a study of an Australian cohort^6^) may be explained by, for instance, (a) rare coding variant alleles not genotyped or imputed in this study, (b) additional extragenic variation affecting *MC1R* expression, (c) dominant action of specific alleles, (d) variation in other genes in the same or a parallel pathway or (e) misreporting of hair colour.

**Fig. 2:**
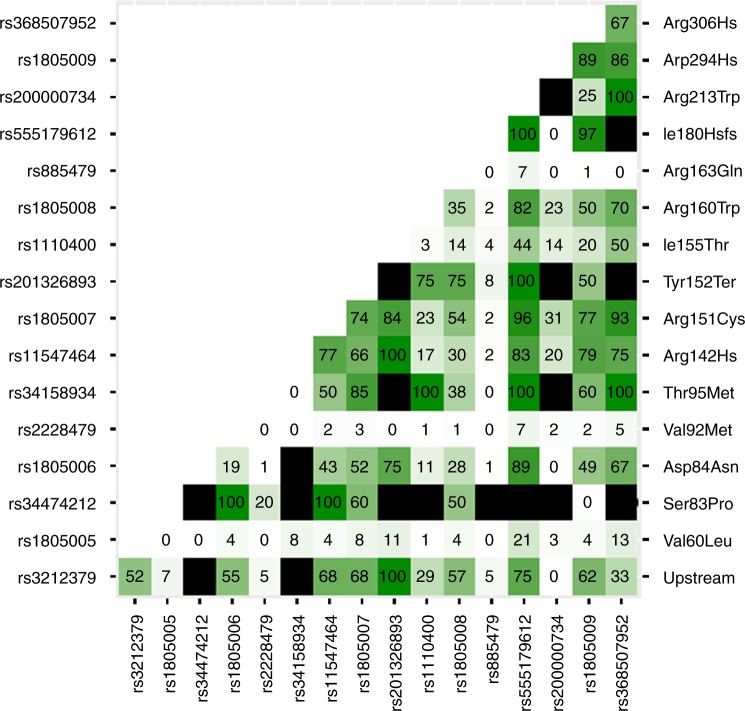
Penetrance matrix of *MC1R* coding variants. Combinations of all coding variants plus the non-coding variant rs3212379, located close to the 5′ end of *MC1R*. Depth of shading in green indicates the % of the genotype with red hair, also indicated by the rounded numbers. Cells filled in black have no data. Full data are in Supplementary Table 2

It is well established that different *MC1R* coding variants have different penetrance with respect to red hair (termed “R” and “r” for high and low penetrance)^6^. With this very large cohort we are able to more precisely quantify the degree of penetrance of each allele, whether as homozygotes or in combination with any other allele. (Fig. 2, Supplementary Table 2). Similar to others we find that penetrance of missense variants ranges from less than 1% as homozygotes (V60L, V92M) to over 90% (D294H). Given the large odds ratios (OR) we consider the three newly identified variants to be high penetrance alleles. We also calculated the minor allele frequency and the OR for red hair for all analysed variants (Table 2). When analysed without conditioning, the three “r” variants have ORs less than 1, as previously described^18^. However, when conditioning on multiple high penetrance variants, the OR for V60L is >3 indicating its effect occurs primarily in combination with other alleles.

**Table 2:** MC1R variants

Variant	A1	Consequence	MAF	OR (initial)	Classification
rs3212379*	T	Upstream	0.010	5.28	Strong-R
rs1805005	T	V60L	0.121	0.32	Weak-r
rs34474212	C	S83P	7×10^−^^5^	5.06	Strong-R
rs1805006	A	D84E	0.009	2.97	Strong-R
rs2228479	A	V92M	0.099	0.11	Weak-r
rs34158934	T	T95M	3×10^−4^	5.52	Strong-R
rs11547464	A	R142H	0.007	4.17	Strong-R
rs1805007	T	R151C	0.105	10.98	Strong-R
rs201326893	A	Y152X	2×10^−^^4^	8.12	Strong-R
rs1110400	C	I155T	0.009	1.14	Weak-r
rs1805008	T	R160W	0.091	4.16	Strong-R
rs885479	A	R163Q	0.040	0.16	Weak-r
rs555179612	TC	I182Hfs	0.002	8.75	Strong-R
rs200000734*	T	R213W	6×10^−4^	2.07	Strong-R
rs1805009	C	D294H	0.022	5.21	Strong-R
rs368507952*	A	R306H	3×10^−4^	7.42	Strong-R

MC1R variants with MAF (UK10K), the initial GWAS odds ratio (before conditioning) and the classification in strong and weak alleles according to the Penetrance Matrix. The three newly identified red-hair alleles are marked with an asterisk (*)

### Additional red hair colour-associated loci

**Fig. 3:**
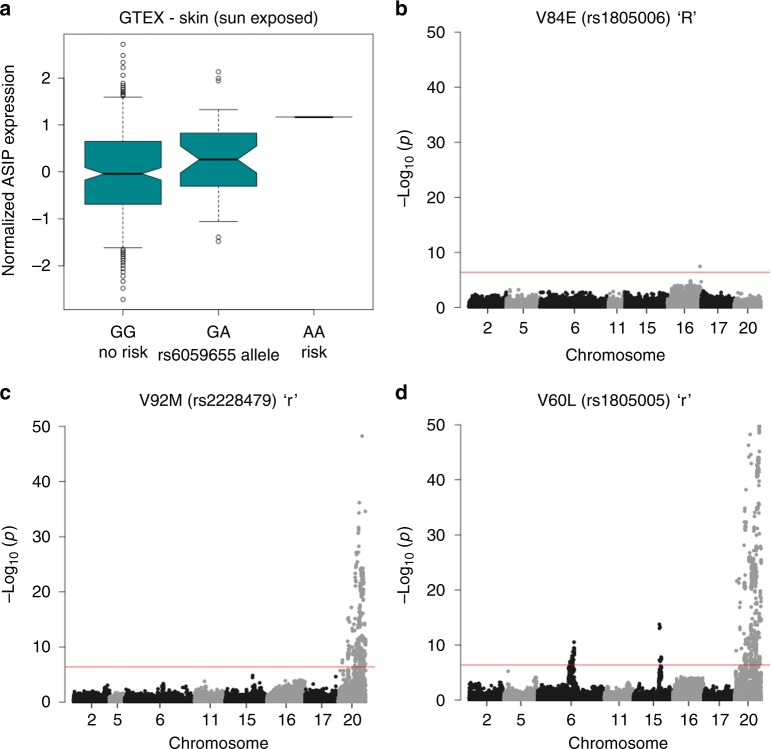
Gene expression variation at ASIP and epistatic interactions with MC1R variants. **a** Gene expression data from GTex of *ASIP* in sun-exposed skin, ordered by genotype at rs6059655 and normalised to the homozygous no-risk genotype (GG). Boxplot indicates the median expression of ASIP, and the error bars indicate 95% of the data in 320 individuals, 277 with no-risk allele, 42 with middle risk allele and 2 with the high risk allele. **b−d** Epistatic interactions between *MC1R* coding variants and other red-hair-associated loci. **b** The high penetrant allele D84E shows no *trans* interactions, **c** the low penetrant allele V92M shows interactions at *ASIP*, **d** the low penetrant allele V60L shows interactions with *ASIP*, *HERC2/OCA2* and *PKHD1*

In addition to the associations around *MC1R* on chromosome 16, we observe 8 additional associations at genome-wide significance (Supplementary Table 1). Statistical fine-mapping of causal SNPs (PICS)^19^ in some cases indicated a single likely casual variant, whilst in others one of more than 50 variants could potentially be the causal SNP. We find a previously unreported association at rs276645354, at which the minor allele reduces the probability of red hair. This variant lies less than 2 kb from transcriptional start site (TSS) of *POMC*, which encodes α-MSH, the agonist of MC1R. Increased expression of *POMC* is likely to promote melanogenic signalling and thus dampen the effect of those MC1R variants which have some, albeit reduced, signalling activity. A single variant in an intron of *RALY*, located 5′ of *ASIP*, the gene encoding the inverse agonist of MC1R, was associated with red hair. This variant, rs6059655, is also an expression QTL (eQTL) for *ASIP* expression in skin, with the red-hair-associated allele showing higher mean expression levels^20^ (www.gtexportal.org) (Fig. 3a). We suggest that variants that increase *ASIP* expression in the skin or hair follicles lead to greater competition with α-MSH for melanocyte MC1R binding, antagonising melanogenic induction and increasing the pheomelanin in melanocytes.

We find a variant in *HERC2* associated with a decreased probability of red hair. It is well established that variants in *HERC2* alter transcription of the neighbouring pigmentation gene *OCA2* which is additionally associated with blue eyes and blonde hair colour^12,21–24^. Recessive mutations in *OCA2* result in albinism. It is possible that varied expression of *OCA2* modifies the effect of reduced signalling though variant MC1R, such that red hair colour is not expressed.

An association is also seen in the *TSPAN10* gene, also known as oculospanin, which is highly expressed in melanocytes and retinal pigment epithelium. The lead SNP lies in strong LD (*r*^2^ = 0.995) with a non-synonymous variant (rs6420484; Y177C) affecting a conserved amino acid. This association signal is also in moderate LD (*r*^2^ ~ 0.4, *D′* ≥ 0.95) with a previously reported association with increased hue-saturation of eye colour, which corresponds to darker eyes, in a Dutch cohort^25^. Previous targeted knockdown of the murine *Tspan10* mRNA resulted in reduced melanocyte migration in a trans-well migration assay^26^, indicating this gene may be a good functional candidate as a novel hair colour gene.

### Epistasis between alleles at MC1R and other loci

Detecting epistasis in complex traits is challenging. Epistatic effects are believed to be much smaller than main effects, which are typically already very small in the case of polygenic traits. However, due to the large effects of some genetic variants on hair colour this might be a more tractable model to detect epistasis. We tested all associated genetic variants from our analysis of red hair colour against each of the *MC1R* coding variants, including the known “R” and “r” *MC1R* red hair colour alleles, by constructing a logistic regression model whilst correcting for relevant covariates (see Methods). At a *P* value of 3.9×10^−7^ (i.e. 0.05/128,205, the number of statistical tests performed), we found consistent epistasis signals between *MC1R* variation and a ±1.5 MB region surrounding rs6059655, which is the hair colour-associated *ASIP* eQTL SNP (Fig. 3b, d, Supplementary Figure 2, Supplementary Table 3). We also detect epistasis between both rs1805005 (V60L) and rs1805008 (R160W) and the *HERC2/OCA2* region. It has been noted previously that *OCA2* variation affects the penetrance of the weaker red hair alleles of *MC1R*^6^. Finally, we also find evidence of epistasis between V60L and *PKHD1* on chromosome 6. The magnitude of the UK Biobank cohort has allowed the identification of hitherto unknown epistatic interactions contributing to red hair.

### Genome-wide association analysis of blonde hair colour

**Fig. 4:**
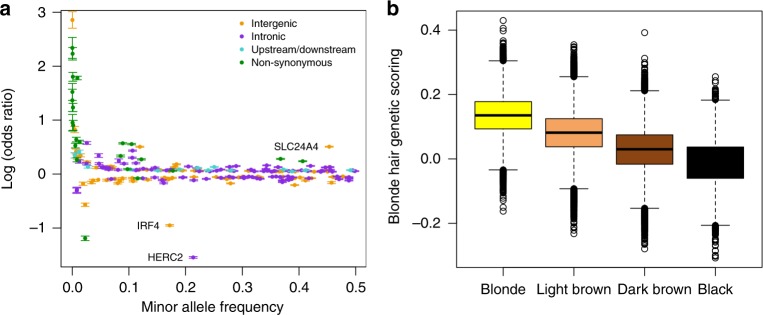
Odds ratio and minor allele frequency for blonde hair and polygenic phenotype scores. **a** Plot of minor allele frequency of blonde hair-associated variants vs. log of the odds ratio for blonde hair. Variants are colour-coded for annotation; intergenic (yellow), intronic (purple), 2 kb upstream or 500 bp downstream (cyan), non-synonymous coding (green). Error bars indicate 95% confidence intervals in OR, according to the logistic model calculated with blonde vs. brown and black hair colour (blonde = 39,397, non-blonde = 283,920). **b** Genetic scores derived from all lead variants from blonde vs. brown plus black hair colour, assuming an additive genetic model. The line in the boxplot indicates the median value and the error bars the 95% of the data (blonde: 39,397 individuals, light brown: 141,414 individuals, dark brown: 127,980 individuals and black: 14,526 individuals). Colours yellow, light brown, dark brown and black match the hair colour analysed

Whilst red hair is essentially a Mendelian trait modified by additional loci, the genetic architecture of blonde hair colour is concordant with a polygenic trait. We performed a genome-wide association analysis comparing blonde hair to combined brown and black hair-coloured individuals. Following conditional association testing to uncover additional signals of association, we discover 213 lead variants associated with blonde hair colour (Fig. 1b, Supplementary Table 4, Supplementary Figure 1b). In many cases multiple signals of association are found close to the same genes. This could be a result of multiple, independent associations (as is the case for *MC1R*, for example). Alternatively some or all signals may each be correlated with the same variant that has been neither genotyped nor imputed. Many signals of association are close to, or within, previously known pigmentation genes from both human and model organism studies These allelic effects span a spectrum of OR and minor allele frequencies consistent with many other phenotypes with an underlying polygenic architecture^27,28^ (Fig. 4a). Using probabilistic association of causal SNPs (PICS)^19^ on these independent variants we are able to find 64 which were likely to be to a single candidate causal SNP, amongst which are 23 coding variants (7of which lie within *MC1R*). Several variants notably stand out, which have been previously associated with a variety of pigmentation related traits in humans (including *SLC24A4, HERC2/OCA2, SLC45A2, TYR, TYRP1, EDNRB*), some of which have been specifically linked to alterations in transcriptional regulation (*IRF4* and *KITLG*)^29,30^.

Genes associated with red hair colour, in particular *MC1R*, are also identified in our blonde hair analysis. Although 93% of individuals with red hair carry two *MC1R* variants, these make up only 15% of people who carry two MC1R variants. The majority of people with two variants have blonde (15%) or light brown hair (41%). The proportion of individuals with blonde hair decreases with one or no variants whilst the proportion with dark brown and black increases (Supplementary Table 5). We show the incidence of different hair colours on each combination of *MC1R* variants in Supplementary Figures 3−6.

Given the observed differences in red and blonde hair frequency between males and females, we performed association analyses separately for each gender. Directly comparing the ORs of all significantly associated variants for males and females we find a strong correlation between the sexes (Supplementary Figures 7−8).

We compared our results for both red and blonde hair with those of Hysi et al.^15^. The variants we identify correspond to 163 distinct genes, of which 93 are also reported by Hysi et al. Conversely, they report 137 significantly associated lead variants, 23 of which we did not analyse because they did not pass our quality control. Of the remaining 114, only 73 show a significant association in our study. However, to better compare the results we looked for significant associations within fixed genomic distances from the 137 of Hysi et al. Within 10 kb of their associated variants we find 93 associations, and within 100 kb we find 100 (Supplementary Table 6). Hence 43% of the genes we identify are novel, and we find 73% of those found by Hysi et al.

### Genome-wide association analysis of brown hair colour

Based on the large number of associations with blonde hair, we hypothesised that hair colour may lie on a continuous genetic spectrum from black to blonde through brown hair. Thus, we might expect to observe a subset of the blonde-associated variants associated with brown hair. Following both primary and conditional analyses we find 56 lead variants associated with brown vs. black hair (Fig. 1c, Supplementary Figure 1c, Supplementary Table 7), 28 of which are the same associated variant with blond hair and with the same direction of effect. Of the remaining lead variants 23 identify the same genes seen in our blonde hair analysis, and further 3 are associated with red hair. Of the two novel genes, *KRT31* lies within a large locus encoding multiple keratin genes, in which we also observe associations with blonde hair. Only *PIGU* does not have a significant association with other hair colours, although we observe an association with another member of the same gene family, *PIGV*, suggesting that paralogous genes may be associated with hair colour differences. Additional GWAS of light brown and dark brown hair colours as expected identify more associations when light brown is compared to dark brown/ black than when brown is taken as a single category. Fewer associations are seen with dark brown alone vs. black (Supplementary Figures 9 and 10).

### Polygenic phenotype scoring

To test the hypothesis that the genetic basis of hair colour is polygenic and that hair colour falls on a continuum as a genetic trait, we constructed a polygenic score for hair colour. Specifically we constructed a blonde hair colour polygenic phenotype score by taking the variants that reached genome-wide significance in the blonde vs. brown and black hair colour conditional analysis (5×10^−8^), as a linear combination of the allele-weighted regularised logistic regression coefficients. We found that self-reported black, dark brown, light brown and blond hair lie on an approximately linear spectrum (Fig. 4b). We confirmed the same pattern across hair colours in two groups of individuals excluded from all previous analyses; related individuals (Supplementary Figure 11a) and individuals with European, but non-British ancestry (Supplementary Figure 11b).

Additionally we calculated the SNP heritability of the different hair colours in the Biobank cohort (Supplementary Table 8). We estimate the SNP heritability of red hair to be 0.403, blonde as 0.301 and brown as 0.234. Removing the MC1R-associated variants on chromosome 16 results in a residual model for red hair with SNP heritability of just 0.018; *MC1R* therefore explains 73% of the observed red hair heritability. Removing all variants associated with red hair, we find a heritability estimate of 0.041 which indicates that the identified loci explain ~90% of the heritability of red hair. Performing the equivalent analysis for blonde hair shows that the identified loci account for 73% of the SNP heritability, and for brown hair the identified loci account for 47% of the SNP heritability.

### eQTL

In order to aid the interpretation of our GWAS and identify functional hypotheses, we tested associated variants for statistical colocalisation with eQTL signals from skin biopsies in the GTex^20^ (Supplementary Tables 9−14) and TwinsUK cohorts^31^, (Supplementary Tables 15−17). We were able to link 37 variants with *cis* eQTLs with high probability (posterior probability > 0.8). Among the variants with the highest probability are at *RALY*, upstream of *ASIP* as noted above and in the first intron of *TSPAN10* for red hair. Most of the eQTLs are associated with gene expression at considerable distance and often with several genes. Among the most significant eQTLs, across all three datasets, are several missense variants in *MC1R*, which are independently linked to expression changes in multiple genes located up to several hundred kb from *MC1R*. These may well be synthetic associations reflecting weak LD and the unusual behaviour of this segment of the genome. Whist the colocalisation of hair colour association signals with skin tissue *cis*-eQTLs may appear promising, they are at best a strong indication of biological effect and will require extensive further hypothesis testing to establish any role in determining pigmentation.

### Hair colour loci are enriched for regulatory features

To understand the transcriptional regulatory mechanisms that might underpin the observed genetic associations with hair colour, we examined the potential for these variants to affect the chromatin landscape in cell types relevant to pigmentation. Specifically, we tested histone tail modifications associated with gene activation or repression and with chromatin accessibility (DNase I hypersensitive sites) in melanocytes, keratinocytes, fibroblasts and other cells. Additionally, the proximity of several association signals to core promoter regions raises the possibility of alterations to TSS and pigmentation cell-specific regulatory factors, i.e. the melanogenesis master regulator MITF.

Using a permutation-based approach (GoShifter)^32^, we tested each annotation in each cell type where data were available (Table 3). We find statistical evidence of enrichment of pigmentation-associated genetic variation overlapping histone marks of both gene activation (H3K4me3) in melanocytes and repression (H3K9me3, H3K27me3) in melanocytes, fibroblasts and keratinocytes. In addition there is enrichment of MITF binding sites in melanocytes and TSS in iris pigmentation cells. These associations give strong support to the notion that we are able to identify functional elements altered by genetic variation.

**Table 3:** Chromatin enrichment

Cell type	Annotation	*P* value	Δenrichment
Melanocyte	H3K27ac	NS	0.00317
	H3K27me3	NS	0.00460
	H3K36me3	NS	−0.00576
	H3K4me1	NS	−0.02505
	H3K4me3	0.00054	0.01526
	H3K9me3	0.00054	0.00921
	DHS	NS	−0.01382
	TSS	NS	−0.00259
	MITF	0.0009	0.01497
Keratinocyte	H3K27ac	NS	0
	H3K27me3	0.0453	0.00749
	H3K36me3	NS	−0.02160
	H3K4me1	NS	0.00605
	H3K4me3	<10^−^^4^	0.02418
	H3K9me3	NS	−0.01785
	TSS	NS	−0.00432
Fibroblast	H3K27ac	NS	0.00346
	H3K27me3	<10^−4^	0.02246
	H3K36me3	NS	0.00461
	H3K4me1	NS	−0.01180
	H3K9me3	0.0038	0.00720
	DHS	NS	−0.02332
Iris pigment	TSS	<10^−4^	0.00519
Dermal fibroblast	TSS	NS	−0.00259
Skin fibroblast	TSS	NS	−0.00086
Skin tissue	TSS	NS	−0.00375
Embryoid melanocytic induction	TSS	NS	−0.00230
Hermes3A	MITF	NS	−0.00374

Enrichment for cell type-specific annotations, using GWAS loci from all hair colours. The Δenrichment column represents the change in enrichment (nsnpOverlap/allsSnps) between our dataset and the median of the 10,000 permutations

### Enrichment for skin and hair genes

To further aid the interpretation of our GWAS findings, and identify shared biological pathways related to pigmentation determination, we took all of the blonde hair lead variants overlapping genic regions extending 2 kb upstream of the TSS and 500 bp downstream of the 3′ end. If no genic region overlapped the lead SNP, then we used the two closest genes within 500 kb (Supplementary Table 18). These candidate genes were then used as input to test for enrichment in known pigmentation phenotypes, utilising the MouseMine database^33^. We identified ~200 orthologous mouse genes in the database, which we analysed for site of expression and mutational phenotypes. Of the 172 genes with expression data, 89 were expressed in the skin (*P* = 1.3×10^−9^) (Supplementary Table 19). One hundred and thirty-two genes had mouse mutant phenotype data and of these, 50 had an integument phenotype (affecting the skin and skin appendages) (*P* = 5.2×10^−^^7^). Not surprisingly, 18 of these affected pigmentation, but we unexpectedly found that 70% affect primarily skin, hair or other skin appendages rather than pigmentation (Table 4).

**Table 4:** Phenotype of mouse mutants at candidate genes

Phenotype	
***Skin/skin appendage***	***Pigmentation***
*Alx4*	*Dct*
*Areg*	*Edn3*
*Asb*	*Ednrb*
*Bmp7*	*Frem2*
*Ccnd1*	*Kitl*
*Cdkal1*	*Mc1r*
*En1*	*Mitf*
***Errfi1***	*Mkln1*
*Fgf5*	*Oca2*
*Fosl1*	*Pax3*
*Foxe1*	*Plk2*
***Fras1***	*Plxnb2*
*Frem2*	*Slc45a2*
*Grm5*	*Tyr*
*Hdac4*	*Tyrp1*
***Hoxc13***	*Xpa*
*Il6*	*Zmiz1*
*Kat6a*	
*Krt33a*	
***Krtap17-1***	
*Lef1*	
***Lgr4***	
*Lhx2*	
*Map3k1*	
*Msx2*	
*Ndufs4*	
*Ovol1*	
***Padi3***	
*Ppm1a*	
*Rspo2*	
*Sp6*	
*Syne2*	
*Twist2*	
*Xpa*	

Skin/skin appendages refers to all skin phenotypes (skin, hair, teeth, sweat glands, mammary glands) except pigmentation. Genes in bold are orthologues of genes identified as affecting hair shape variation

Follicular melanocytes, keratinocytes and dermal papilla cells have mutual interactions; the dermal papilla signals to melanocytes with ASIP, the melanocytes transfer melanin granules into the keratinocytes. Perturbations of these interactions could affect the amount and type of melanin delivered to the hair. Furthermore, variation in growth rate could impact the effectiveness of melanin transfer. Recent GWAS have identified 14 loci associated with hair shape variation^34^. Remarkably, we have identified seven of these, *ERRFI1*, *FRAS1*, *HOXC13*, *PADI3*, *KRTAP*, *PEX14* and *LGR4* as affecting blonde/non-blonde hair colour (*P* = 1×10^−^^11^, Fisher’s exact test). In addition, the refractive and reflective properties of individual hairs may affect perceived colour^35^ and there is evidence that different coloured hairs have different morphology. Vaughn et al. have demonstrated a strong inverse correlation between the lightness of hair colour and the diameter of the shaft; blonde hair is thinner than dark^36^.

In summary, the very large dataset provided by UK Biobank has enabled us to dissect the complex genetic nature of hair colour. This forms the foundation for functional analysis linking genetic variation to phenotype and exploring the cellular interactions between melanocytes and other cells in the hair follicle.

## Methods

### Study participants

Study individuals were derived from the UK Biobank cohort that consists of 502,655 individuals aged between 40 and 69 years at recruitment, ascertained from 22 centres across the UK between 2006 and 2010. The study was approved by the National Research Ethics Committee, reference 11/NW/0382, and informed consent was obtained from all participants as part of the recruitment and assessment process. From these, we analysed 343,234 unrelated individuals, who reported their background as “British” and with similar ancestral backgrounds based on PCA^16^.

### Genotype quality control

Variants included in the analysis were autosomal SNPs present in HRC imputation file, with a *χ*^2^ *P* value for the Hardy−Weinberg equilibrium >10^−10^ (calculated using plink and unrelated white British individuals), a call rate >0.95 in unrelated white British individuals, a UKBB imputation score > 0.9 and an MAF >10^−^^4^. The number of SNPs analysed after quality control is 9,154,080.

### Phenotype quality control

Self-reported hair colour (before greying occurred) for UK Biobank participants was selected from one of eight possible categories: “Blonde”, “Red”, “Light brown”, “Dark brown”, “Black”, “Other”, “Prefer not to answer”. The number of individuals in each category are: Red: 15,731, blonde: 39,397, light brown: 141,414, dark brown: 127,980, black: 14,526, other: 4186, Do not know/Prefer not to answer: 650. Individuals with missing data (“Prefer not to answer”, “Other”, “Do not know”) were excluded from all the analysis. For the red hair colour against brown and black hair colour, the blonde individuals were removed (red = 15,731, non-red = 283,920) and self-reported red hair individuals were removed from the blonde against brown and black analysis (blonde = 39,397, non-blonde = 283,920). For the comparison of brown vs. black, light and dark brown individuals were combined and compared to black hair individuals (brown = 269,394, black = 14,526).

### Genome-wide association and conditional analysis

Following individual and genotype-level QC, a logistic regression model was used to regress presence/absence of each hair colour on bi-allelic variant genotype assuming a (log) additive model, adjusting for the first 15 axes of variation from the PCA and genotyping batch (Eq. (1)).

1 $${\mathrm {logit}}\left( Y \right) = \beta _0 + \beta _{{\mathrm {gtest}}} + \beta X + \varepsilon.$$

Conditional analyses used the same multivariate logistic regression model above, with the addition of the lead SNP (denoted by *βA*_glead_) from each signal of association [2].

2 $${\mathrm {logit}}\left( Y \right) = \beta _0 + \beta A_{{\mathrm {glead}}} + \beta _{{\mathrm {gtest}}} + \beta X + \varepsilon.$$

In the chromosomes where more than one signal of association was apparent (lead SNP *p* ≤ 5×10^−8^), subsequent rounds of conditional analysis were performed, adding each new lead SNP as an extra conditioning SNP (denoted by *βA*_glead_, where *A*_glead_ in Eq. (2) is a matrix of individual lead SNP genotypes). To start this stepwise procedure, we first removed the SNPs with *P* value > 0.1 in the initial GWAS, and then performed the conditional analysis until no single SNP association exceeded *p* ≤ 5×10^−8^. Then, SNPs with an initial *P* value > 0.1 were included again and the conditional analysis continued until no SNPs exceeded *P* ≤ 5×10^−8^. There were just three SNPs that were ~10^−8^ in blonde hair colour, and only one extra round of conditional analysis was needed. PLINK v1.9 was used for the regression analysis and Manhattan and Q-Q plots were generated using the R package qqman^37^ and ggplot2.

### Penetrance

The penetrance of allelic combinations was calculated by summing the number of individuals of a particular hair colour with a given allelic combination over the sum of all individuals (all hair colours) with that allele combination. The calculation and plot were made using the .ped and .map plink files, and plotting was performed using ggplot2 in R.

### Probabilistic inference of causal SNPs

We implemented the probabilistic inference of causal SNPs (PICS)^19^ which takes into account the strength of association of the lead genetic variant and the LD structure in the fine-mapping interval to calculate the posterior probability that a particular genetic variant is the causal variant given that another variant is the lead variant. PICS calculates the conditional posterior probability *P*(*B*^causal^|*A*^lead^), that is the probability that SNP *B* is the causal variant given SNP *A* is the lead SNP. Under the assumption that effect sizes for non-causal SNPs are drawn from a Normal distribution, *N*(*μ*,*σ*^2^), Farh et al. derive from empirical data the sample standard deviation, *σ*_s_, and the expected mean association signal, *μ*_s_, which scales linearly with the LD to the true causal SNP.

3 $$\sigma _{\mathrm s} = \sqrt {1 - r^k} \frac{{\sqrt {{\mathrm {index}}P} }}{2},$$

4 $$\mu _{{\mathrm s}} = r^2 \times {\mathrm {index}}P,$$

where index*P* is the −log_10_*P* of association of the causal SNP for that locus, which is taken to be the lead SNP for that fine-mapping interval. The calculated posterior probabilities were used to construct 95% credible intervals, defining the resolution of fine-mapping over a given genomic interval.

### Epistasis

Variants within 3.0 Mb of the index SNP (±1.5 Mb either direction) for each signal of association were selected for epistasis analysis. Explicit testing of interactions between *MC1R* non-synonymous variants and red hair colour-associated loci was performed by comparing the following full (5) and reduced (6) logistic regression models:

5 $${\mathrm {logit}}\left( Y \right) = \beta _0 + \beta _{{\mathrm g}A} + \beta _{{\mathrm g}B} + \beta _{{\mathrm g}A{\mathrm g}B} + \beta X + \varepsilon,$$

6 $${\mathrm {logit}}\left( Y \right) = \beta _0 + \beta _{{\mathrm g}A} + \beta _{{\mathrm g}B} + \beta X + \varepsilon,$$

where *β*_0_ is the model intercept, *β*_g*A*_ is the regression coefficient for SNP *A*, *β*_g*A*_ is the regression coefficient for SNP *B* and *β*_g*A*g*B*_ is the joint regression coefficient for SNPs *A* and *B*, i.e. the interaction term. Covariates were included in the model, denoted by the model term *βX*. For instance, population structure confounding may be accounted for by including *N* principal components in the model as covariates. Significance testing was performed using a likelihood ratio test, comparing model (5), with the reduced model (6) that omitted the interaction term, *β*_g*A*g*B*_. Epistasis testing was performed using cassi (see URLs) using the --lr command. Due to complex LD structure around the *MC1R* region it was necessary to remove variants in LD (*r*^2^ ≥ 0.1) with the target variant in each case. Additionally, SNP alleles that co-occur on the same haplotypes, but are in imperfect LD, may generate the false impression of interactions, Wood et al.^38^. suggest including the main effects as covariates to remove these false interactions. Therefore, in the SNPs where there was apparent epistasis in chromosome 16, we corrected for rs34357723 and the MC1R SNPs (Table 2), and if there were still signs of epistasis, we corrected for all the significant SNPs in the red vs. brown and black hair conditional analysis (Supplementary Table 1). Correcting for these SNPs we removed most of the epistasis signals on chromosome 16.

### Hair colour polygenic scoring

In order to calculate the hair colour genetic score we performed least absolute shrinkage and selection operator (LASSO). A penalised logistic regression model was created for blonde colour, including all lead variants for blonde vs. black and brown hair colour. The parameter *λ* was selected using the most regularized model from a ten-fold cross validation, within one standard error of the minimum using the glmnet package in R^39^. The regularized effect estimates were used to create genetic scores for blonde hair colour, assuming an additive genetic model. Non-British (*n* = 44,595) and related (*n* = 64,571) individuals were taken as independent test for the blonde hair genetic scoring. The summed genetic score for each individual was calculated using Plink v1.9.

### Chromatin enrichment

Bed format files were downloaded for epigenetic marks associated with activated or accessible chromatin from the Roadmap Epigenome and ENCODE project websites (October 2017). Files were downloaded for annotations in primary melanocytes, keratinocytes, and fibroblasts included in the Roadmap Epigenome Project. Annotations were H3K4me1, H3K4me3, H3K27ac and DNase I hypersensitive sites (DHS; where available). Primary melanocyte, keratinocyte and other pigmentation cell type transcriptional start site (TSS ± 100 bp) data were downloaded from the FANTOM5 website (October 2017).

Micropthalmia-associated transcription factor is generally considered the master regulator of melanogenesis in melanocytes. Short read data were downloaded from SRA corresponding to MITF ChIP-seq data derived from primary melanocytes and the melanocytic cell line Hermes3A^40–42^. Short sequencing reads were extracted using fastq-dump and assessed for quality using fastQC (see URLs). Reads were processed in order to remove adaptor contamination using trimmomatic and then aligned to GRCh37/hg19 using BWA-aln^43^. As there are no replicates in the ChIP-seq data, peaks were called with MACS2^44^ with an FDR 1%. For each cell type and annotation where more than one sample existed, intervals were merged using Bedtools^45^ intersect intersect for two files and multiIntersectBed for >2 files per cell type. To test for genomic enrichment we used a method that tests for the local enrichment of lead variants and those in LD with genomic features by randomly shifting annotations to generate a null distribution; implemented in GoShifter^32^. LD values calculated from the 1000 Genomes phase 3 EUR reference panel were used in conjunction with the lead variant from each independent signal of association (after conditional analyses). GoShifter was run for 10,000 permutations and enrichment *P* values were adjusted for multiple testing using the Benjamini and Hochberg procedure implemented in the R function p.adjust with a 5% FDR.

### Eqtl analysis

To test for the potential of non-coding association signals to impact on gene regulation, we utilised eQTL summary statistics from the GTex^20,46^ and MuTHER^31,47^ studies in tissues relevant to pigmentation (MuTHER; skin biopsies, GTex; sun-exposed and sun not-exposed skin tissue). Summary statistics for genes that fell within 3 Mb intervals of the lead SNP of each association signal were extracted. We wished to test the explicit hypothesis that the same causal variants underlie each eQTL and our association signal for each hair colour (*H*_A_). Subsequently the null hypothesis is a compound null of no association for either trait (*H*_01_) and the combinations of overlapping but independent eQTL and trait associations (*H*_02_) and an association in one trait/eQTL but not the other (*H*_03_ and *H*_04_). A Bayesian testing framework that tests the set of hypotheses relating to those defined above (H_01−4_ and *H*_A_) was developed by Giambartolomei et al.^48^ and is implemented in the R package coloc. Subsequently, we report the posterior probability for each association signal-gene eQTL signal and the alternative hypothesis *H*_A_ (PP4).

### Hysi et al. comparison

Hysi et al.^15^ report 141 significant SNPs in 123 different loci in a meta analysis of UKBiobank phase 1 and 23andme data. As the Hysi et al. analysis takes hair colour as a quantitative variable (1 = blonde, 2 = red, 3 = light brown, 4 = dark brown and 5 = black), we could not compare the results directly. To compare the results, we took their significant SNPs and then we took the lowest *P* value in our GWAS analyses (red vs. non-red, blonde vs. non-blonde or brown vs. black) and calculated the overlap between their significant SNPs and ours. We also ran a new GWAS taking hair colour as a quantitative variable and compared our results to Hysi et al. Some of the SNPs reported in their analysis do not pass our QC, so they have been reported as missing SNPs (NA). Finally, we calculated the overlap with significant SNPs in our data for different distances (1kb, 5kb, 10kb, 50kb, 100kb, 500kb) around SNPs reported significant in the Hysi et al. paper.

### Genotype by sex interactions

UK Biobank unrelated white British data consist in 184,929 females and 158,957 males. In females 12.7% have blonde hair and 5.2% have red hair, while in males 9.9% have blonde hair and 3.7% have red hair. We performed a GWAS for red vs. non-red (excluding blonde hair individuals) and blonde vs. non-blonde (excluding red hair individuals) for males and females separately. With SNPs significant in at least one of the GWAS results (male or female) we did a linear regression of the OR for each hair colour, to see the different effects of the SNPs in different sexes. Using LD Score^49,50^ we calculated the genetic correlation between males and females for blonde and red hair colours

### Heritability

LD score was also used to calculate SNP heritability for hair colour. To calculate the amount of heritability explained by the significant SNPs we removed these SNPs from the analysis and we calculated the heritability again, the change in heritability is the heritability explained by the SNPs we removed. In red hair individuals, we were also interested in the heritability explained by chromosome 16, so we removed the significant SNPs in chromosome 16 and calculated again the heritability of red hair

### URLs

Gtex: https://www.gtexportal.org/

Cassi: http://www.staff.ncl.ac.uk/richard.howey/cassi/

Twins UK: http://www.muther.ac.uk/

SRAtoolkit: https://github.com/ncbi/sra-tools

fastqQC: https://www.bioinformatics.babraham.ac.uk/projects/fastqc/

Roadmap Epigenomics Project: www.roadmapepigenomics.org/data/

FANTOM5: fantom.gsc.riken.jp/5/

## Electronic supplementary material

Supplementary Information

Peer Review File

Reporting Summary

## Data Availability

Summary statistics can be obtained on request from the authors. The raw genetic and phenotypic data that support the findings of this study are available from UK Biobank, but restrictions apply to the availability of these data, which were used under licence for the current study, and so are not publicly available. Data are, however, available from the authors on reasonable request and with permission from UK Biobank (http://www.ukbiobank.ac.uk). A Reporting Summary for this Article is available as a Supplementary Information file.
