# Supplementary material for: Genome-wide study of hair colour in UK Biobank explains most of the SNP heritability
Source: Nat Commun. 2018 Dec 10;9:5271. doi: 10.1038/s41467-018-07691-z (PMC6288091; doi:10.1038/s41467-018-07691-z)
Supplement: Supplementary file 1 — Supplementary Information [file 41467_2018_7691_MOESM1_ESM.pdf]

Supplementary Information

Genome-wide study of hair colour in UK Biobank explains most of the heritability

Morgan et al.

SUPPLEMENTARY FIGURES:

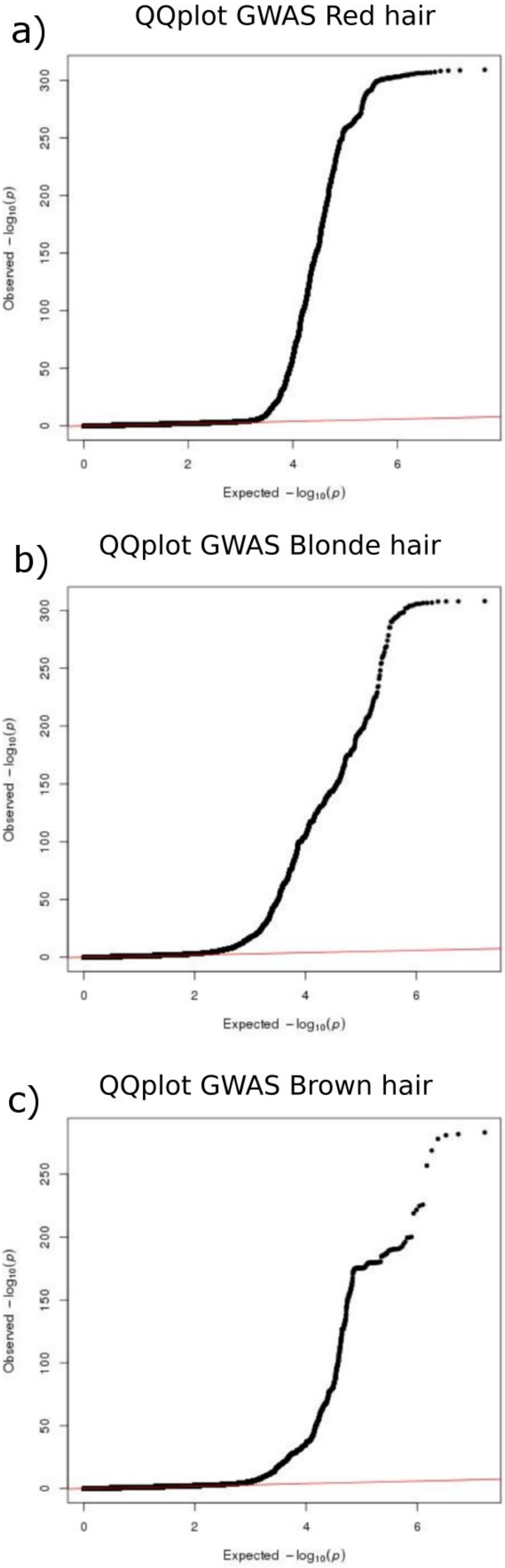

Supplementary Figure 1: QQ plots for the GWAS a) is red versus black plus brown hair colour, b) is blonde versus black plus brown hair colour and c) is brown versus black hair colour.

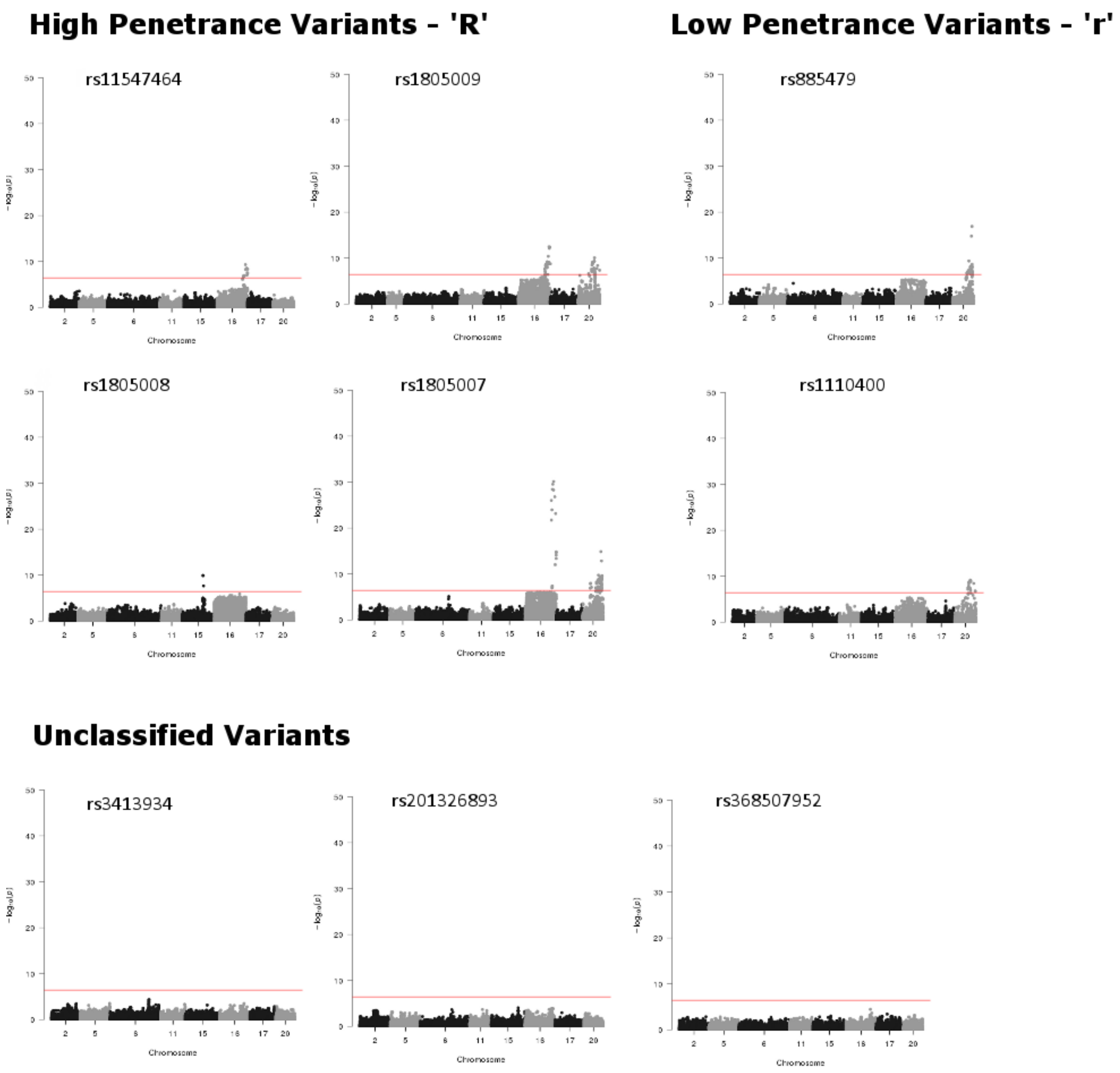

Supplementary Figure 2: Epistasis between *MC1R* variants and non-*MC1R* red-hair associated loci. Each panel represents a Manhattan plot for each MC1R variant tested against all other red-hair associated loci; the variants rsID are indicated above each panel. Variants are grouped based on a previous classification into high ('R') and low ('r') penetrance variants for red hair colour.

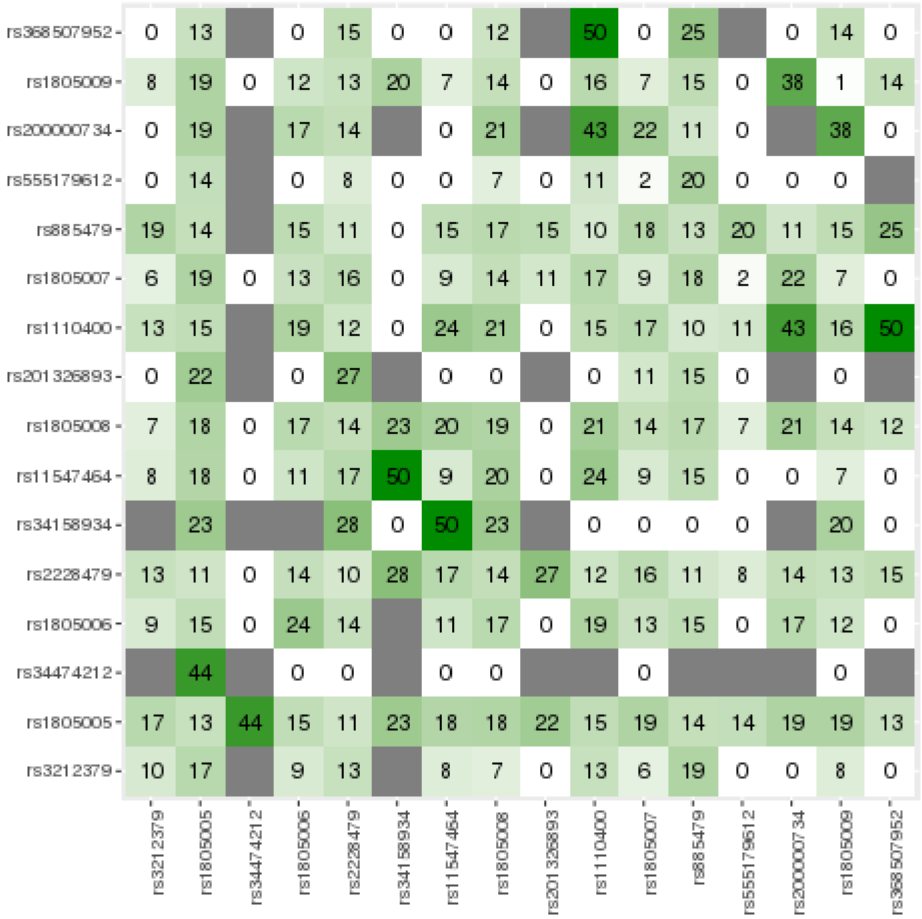

Supplementary Figure 3: *MC1R* penetrance for blonde hair. Penetrance matrix of all *MC1R* coding variants, and a single promoter variant rs3212379, amongst blonde haired individuals. Depth of shading in each cell represents the strength of the penetrance for each allelic combination; the numbers represent the integer percentages. Grey cells are combinations not present in data.

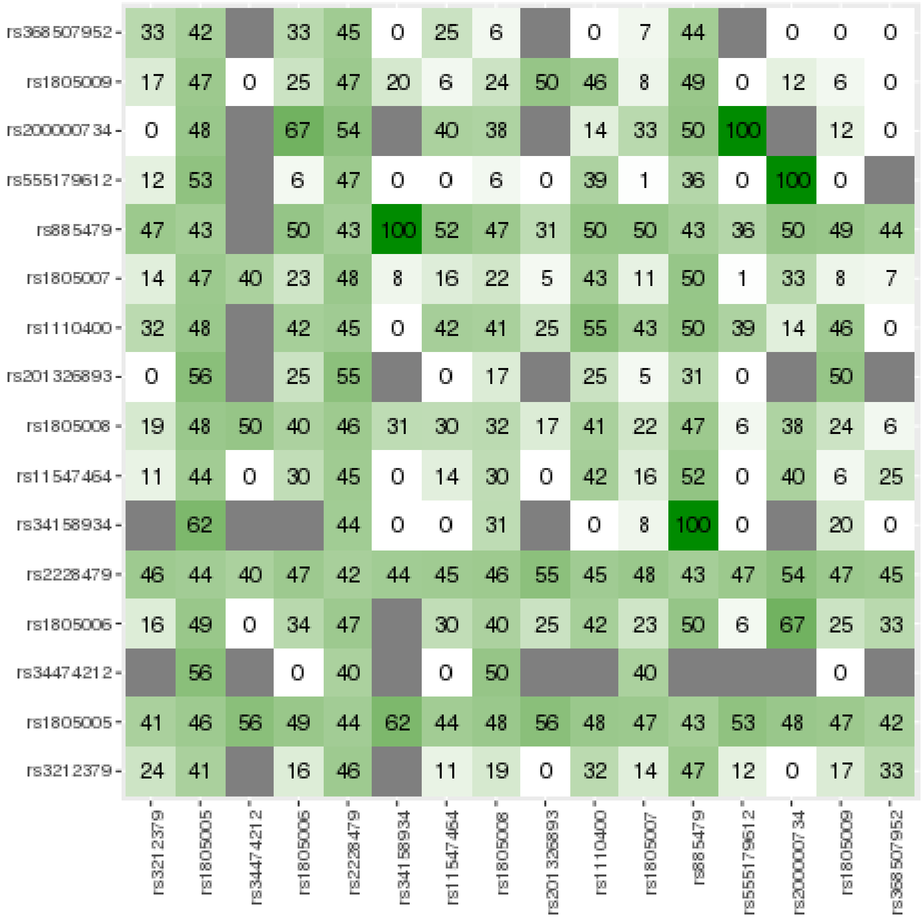

Supplementary Figure 4: *MC1R* penetrance for light brown hair. Penetrance matrix of all *MC1R* coding variants, and a single promoter variant rs3212379, amongst light brown haired individuals. Depth of shading in each cell represents the strength of the penetrance for each allelic combination; the numbers represent the integer percentages. Grey cells are combinations not present in data

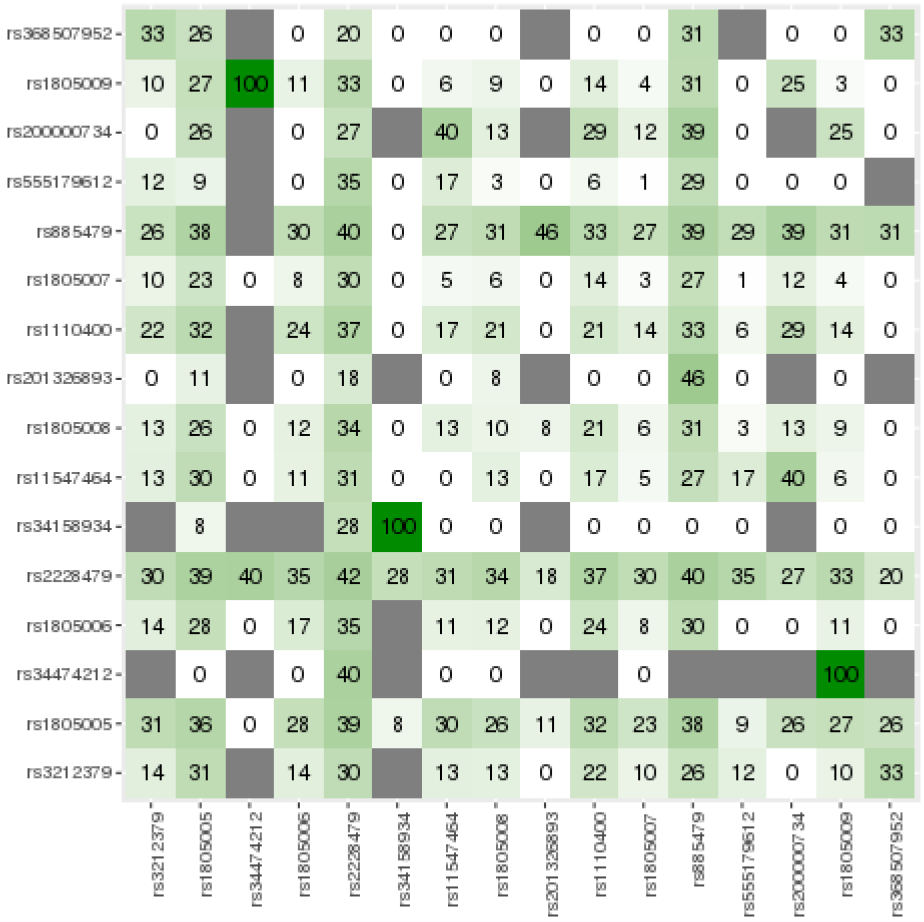

Supplementary Figure 5: *MC1R* penetrance for dark brown hair. Penetrance matrix of all *MC1R* coding variants, and a single promoter variant rs3212379, amongst dark brown haired individuals. Depth of shading in each cell represents the strength of the penetrance for each allelic combination; the numbers represent the integer percentages.

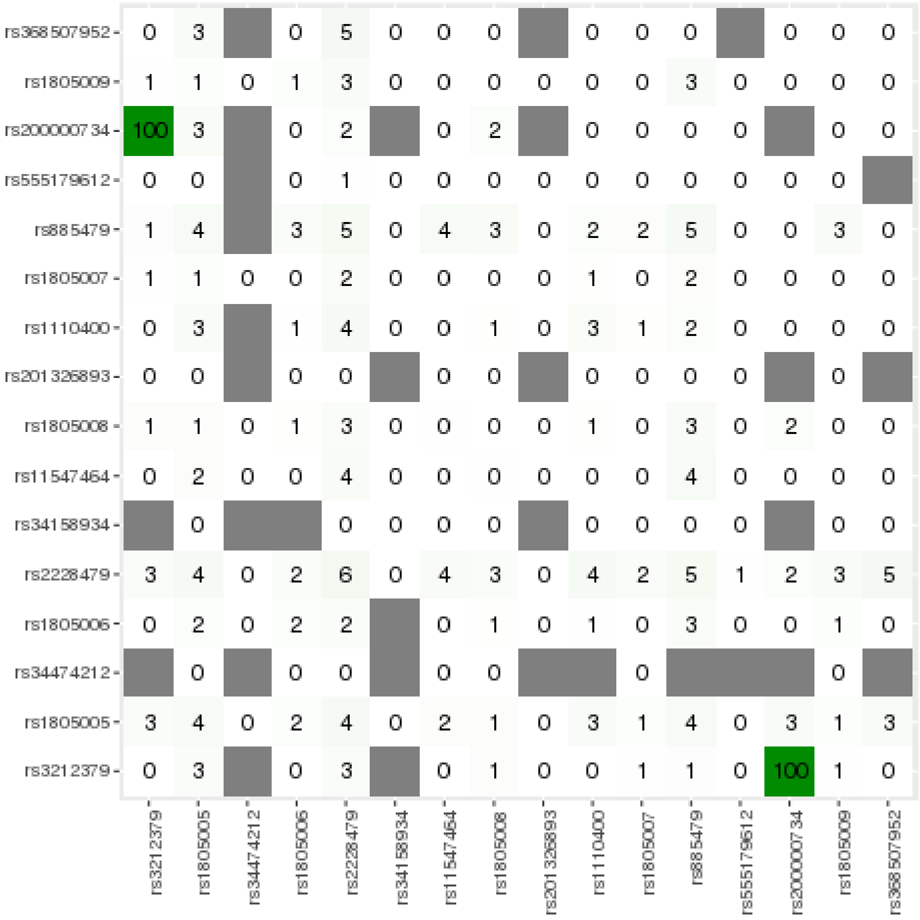

Supplementary Figure 6: *MC1R* penetrance black hair. Penetrance matrix of all *MC1R* coding variants, and a single promoter variant rs3212379, amongst black haired individuals. Depth of shading in each cell represents the strength of the penetrance for each allelic combination; the numbers represent the integer percentages. Grey cells are combinations not present in data

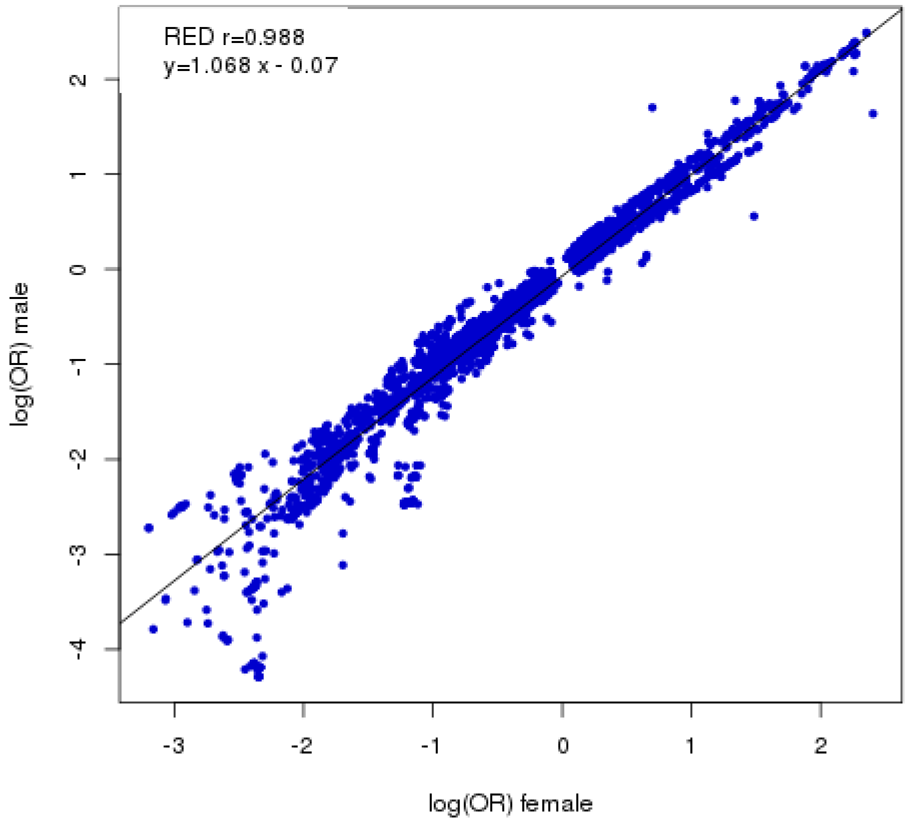

Supplementary Figure 7: Concordance between male and female red hair associations. A scatter plot illustrating the strong correlation between effect size estimates (ORs) between males and females for red hair colour. The genetic correlation for red hair is 0.988, and the slope of the line is ~1.

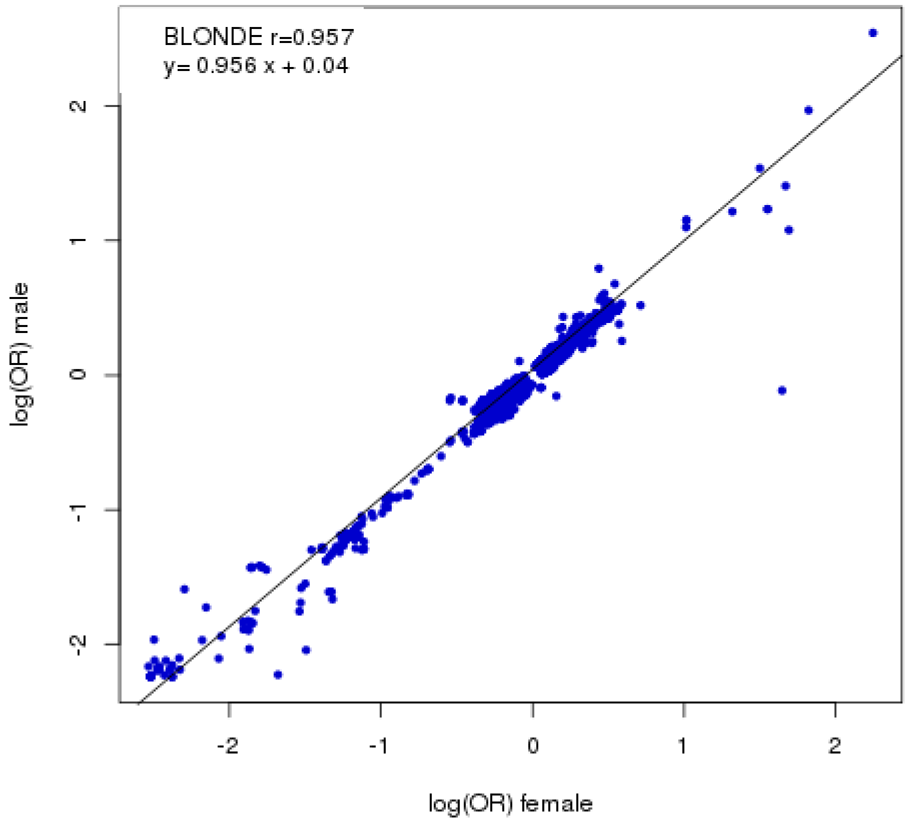

Supplementary Figure 8: Concordance between male and female blonde hair associations. A scatter plot illustrating the strong correlation between effect size estimates (ORs) between males and females for blonde hair colour. The genetic correlation for red hair is 0.957, and the slope of the line is ~1.

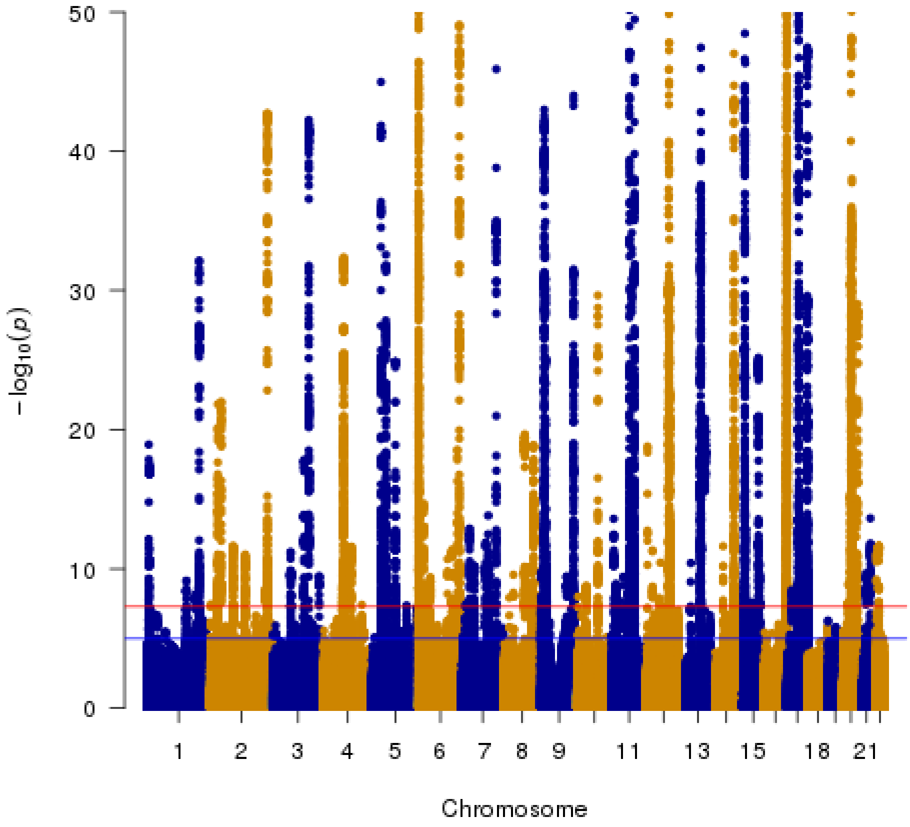

Supplementary Figure 9: A Manhattan plot of a light brown hair GWAS. Individuals with light brown hair were compared to a combined group of dark brown and black hair. The –$\log_{10}$ P-value is plotted (y-axis) for each tested variant, order by chromosome and position (x-axis). Y-axis values are capped at 50 for clarity.

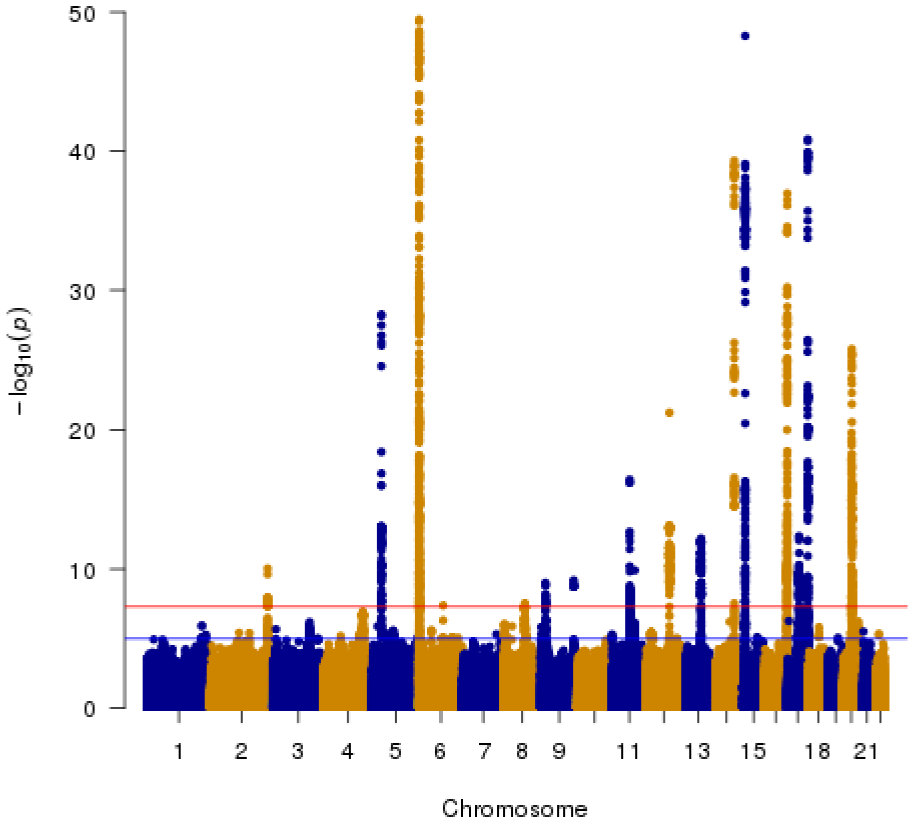

Supplementary Figure 10: A Manhattan plot of a dark brown hair GWAS. Individuals with dark brown hair were compared to those with black hair. The $-\log_{10}$ P-value is plotted (y-axis) for each tested variant, order by chromosome and position (x-axis). Y-axis values are capped at 50 for clarity.

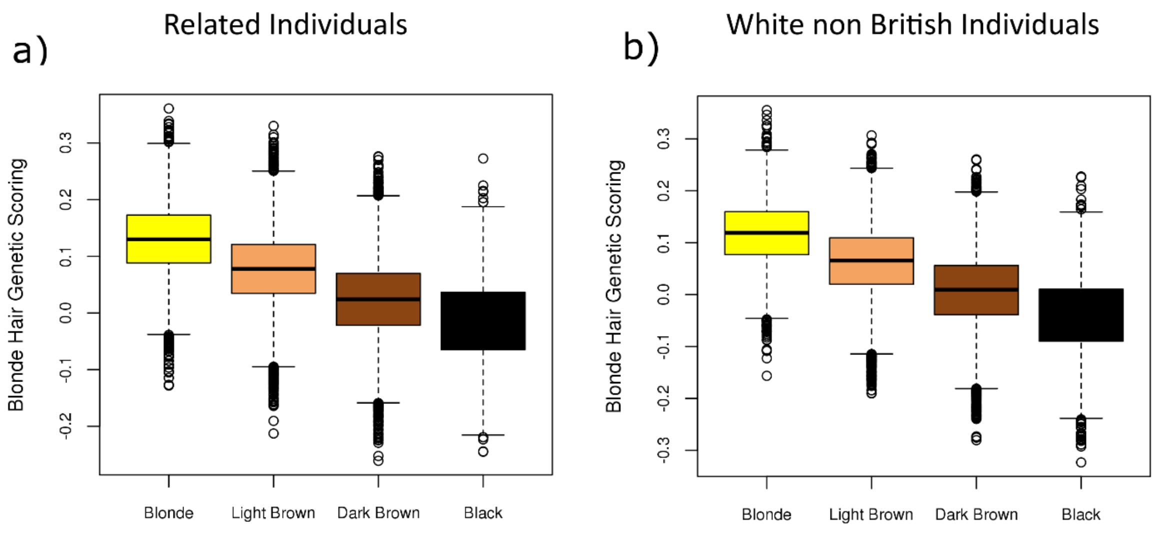

Supplementary Figure 11: Polygenic Phenotype Scores. Genetic scores derived from all lead variants from blonde versus brown plus black hair colour, assuming an additive genetic model, applied to confirmation cohorts within Biobank: 3rd degree relatives or closer individuals (blonde=7327, light brown= 26803, dark brown=23764 and black=2750) in a) and white non-British individuals (blonde=4450, light brown=15923, dark brown=18565 and black=3156) in b). . The line in the boxplot indicates the median value and the error bars the 95% of the data. Colours yellow, light brown, dark brown and black match the hair colour analysed.

Supplementary Table 1: Red hair vs black plus brown GWAS, including analysis following serial conditioning for successive chromosome 16 variants

**Chromosome**	**Locus**	**Variant**	**Minor.allele**	**MAF**	**OR**	**z.statistic**	**p.value**	**Annotation**	**Position**	**Gene2**	**PICS**
2	POMC	rs7664536	G	0.1836	0.905	-6.443	1.17E-10	Upstream 2KB	25393388		57
5	MSX2	rs6876712	T	0.257	1.123	8.914	4.90E-19	intergenic	173976115		10
6	PKHD1	rs9463733	G	0.3195	0.8683	-11.03	2.83E-28	Intron	51722693		2
11	ORAOV1	rs638640	C	0.34	1.078	6.19	6.02E-10	intergenic	69387455		34
15	HERC2	rs1129038	A	0.2158	0.7112	-22.56	3.29E-113	5’ UTR	28356859		1
16	FANCA/SPIRE2	rs3435772	T	0.2745	9.589	154.3	2.25E-308	Intron	89886519		2
17	TSPAN10	rs9747347	T	0.3578	1.12	9.465	2.94E-21	Intron	79606820		14
20	RALY	rs6059655	A	0.1059	1.879	40.1	2.25E-308	Intron	32665748		1
21	SIK1	rs672948	A	0.4145	1.07	5.729	1.01E-08	intergenic	44793448		3
Conditional											
16	MC1R	rs1805007	T	0.1048	10.99	151.6	2.25E-308	missense	89986117		1
16	MC1R	rs1805008	T	0.08918	12.84	127.1	2.25E-308	missense	89986144		1
16	AFG3L1P	rs5758667	A	0.02579	39.43	110.2	2.25E-308	intron	90052987		1
16	MC1R	rs1154746	A	0.007148	37.62	63.74	2.25E-308	missense	89986091		1
16	CDK10	rs1169275	T	0.004391	57.09	58.46	2.25E-308	Intergenic	89743627	CHIMP1A	1
16	MC1R	rs1805006	T	0.01312	22.55	59.31	2.25E-308	missense	89985918		1
16	MC1R	rs5551796	TC	0.001706	231.5	49.89	2.25E-308	missense	89986202		1
16	MC1R	rs1805005	T	0.122	3.255	33.31	2.51E-243	missense	89985844		1
16	MC1R	rs1110400	C	0.01216	9.997	31.23	3.76E-214	missense	89986130		14
16	FANCA	rs1800347	C	0.04798	4.521	31.75	3.11E-221	Intron	89815049		1
16	MC1R	rs3685079	A	0.000416	71.08	20.51	1.86E-93	missense	89986583		1
16	TUBB3	rs2302898	A	0.29	2.456	21.58	2.99E-103	Intron	89998794		1
16	TCF25	rs7247700	C	0.1094	2.554	16.51	2.96E-61	Intron	89950299		5
16	MC1R	rs2013268	A	0.000237	288.8	16.53	2.16E-61	Stop	89986122		1
16	DEF8	rs2009984	T	0.000262	131	15.38	1.98E-56	synonymous	90024048		1
16	MC1R	rs2000007	T	0.000613	32.03	14.86	5.79E-50	missense	89986303		1
16	CHMP1A	rs2014791	T	0.001465	11.37	14.85	6.55E-50	missense	89715813		1
16	MC1R	rs3212350	A	0.419	0.444	-13.52	1.23E-41	upstream	89983554		7
16	CBFA2T3	rs5478590	G	0.003856	3.014	11.51	1.11E-30	intron	88961093		2
16	TCF25	rs4785736	C	0.4818	1.937	10.34	4.85E-25	Intron	89969593		1
16	DEF8	rs1158413	T	0.06782	0.4594	-10.35	4.28E-25	Intron	90029660		1

16	DPEP1	rs5583987	C	0.1065	1.46	7.428	1.10E-13	3 prime UTR	89707493	1
16	TCF25	rs7825896	A	0.007019	3.299	7.433	1.06E-13	Intron	89959888	1
16	FTO	rs1695300	A	0.1621	1.19	7.308	2.71E-13	Intron	54114824	3
16	TCF25	rs1186238	C	0.1331	0.358	-7.286	3.20E-13	Intron	89972532	43
16	FANCA	rs1164691	A	0.1045	0.6388	-7.052	1.77E-12	Intron	89818265	1
16	SPIRE2	rs1205151	A	0.07865	0.5643	-6.667	2.62E-11	Intron	89913008	6
16	DEF8	rs1172046	T	0.0335	3.548	6.549	4.29E-11	3 prime UTR	90032455	1
16	MC1R/TCF25	rs8045560	C	0.4191	7.44	6.221	4.93E-10	Intron	89979494	8
16	FANCA	rs1509245	T	0.006087	2.52	5.911	3.40E-09	Intron	89816815	1
16	TUBB3	rs4586434	A	0.2906	0.353	-5.492	3.92E-08	Intron	89994916	6

	rs3212379	rs1805005	rs34474212	rs1805006	rs2228479	rs34158934	rs11547464	rs1805008	rs201326893	rs1110400	rs1805007	rs885479	rs555179612	rs200000734	rs1805009	rs368507952
**rs3212379**	11/21	63/862	0/0	31/56	24/465	0/0	26/38	231/407	1/1	20/68	322/475	12/227	6/8	0/1	80/128	1/3
**rs1805005**	63/862	25/5112	0/9	46/1133	13/8255	1/13	22/572	328/7536	2/18	13/1063	695/8915	9/4100	27/127	2/58	82/2165	4/31
**rs34474212**	0/0	0/9	0/0	1/1	1/5	0/0	1/1	1/2	0/0	0/0	3/5	0/0	0/0	0/0	0/1	0/0
**rs1805006**	31/56	46/1133	1/1	11/59	12/888	0/0	26/61	231/815	3/4	13/114	514/981	5/423	16/18	0/6	120/243	4/6
**rs2228479**	24/465	13/8255	1/5	12/888	5/3308	0/18	9/456	77/6021	0/11	6/815	182/7135	6/3287	8/108	1/56	33/1745	1/20
**rs34158934**	0/0	1/13	0/0	0/0	0/18	0/1	1/2	5/13	0/0	2/2	11/13	0/5	1/1	0/0	3/5	1/1
**rs11547464**	26/38	22/572	1/1	26/61	9/456	1/2	17/22	138/457	2/2	10/59	356/541	4/211	5/6	1/5	103/130	3/4
**rs1805008**	231/407	328/7536	1/2	231/815	77/6021	5/13	138/457	975/2813	9/12	96/707	3540/6529	62/3088	95/116	12/52	768/1528	23/33
**rs201326893**	1/1	2/18	0/0	3/4	0/11	0/0	2/2	9/12	0/0	3/4	16/19	1/13	1/1	0/0	2/4	0/0
**rs1110400**	20/68	13/1063	0/0	13/114	6/815	2/2	10/59	96/707	3/4	2/66	187/815	16/398	8/18	1/7	40/201	1/2
**rs1805007**	322/475	695/8915	3/5	514/981	182/7135	11/13	356/541	3540/6529	16/19	187/815	2973/4034	80/3485	111/116	16/51	1435/1863	28/30
**rs885479**	12/227	9/4100	0/0	5/423	6/3287	0/5	4/211	62/3088	1/13	16/398	80/3485	3/956	4/59	0/18	10/870	0/16
**rs555179612**	6/8	27/127	0/0	16/18	8/108	1/1	5/6	95/116	1/1	8/18	111/116	4/59	3/3	0/1	35/36	0/0
**rs200000734**	0/1	2/58	0/0	0/6	1/56	0/0	1/5	12/52	0/0	1/7	16/51	0/18	0/1	0/0	2/8	1/1
**rs1805009**	80/128	82/2165	0/1	120/243	33/1745	3/5	103/130	768/1528	2/4	40/201	1435/1863	10/870	35/36	2/8	143/161	6/7
**rs368507952**	1/3	4/31	0/0	4/6	1/20	1/1	3/4	23/33	0/0	1/2	28/30	0/16	0/0	1/1	6/7	2/3

Supplementary Table 2: Matrix of penetrance of red hair phenotype for each genotype combination

Supplementary Table 3: Epistasic Interactions. Tests for interactions between *MC1R* coding variants and non-*MC1R* red-hair associated variants. The lead SNP at the interacting locus is shown with the OR and P-value. NS=not significant

**MC1R**		**Chr6**				**Chr15**				**Chr20**		
**SNP**	SNP2	OR (95% CI)	C.I (95%)	Pval	SNP2	OR (95% CI)	C.I (95%)	Pval	SNP2	OR (95% CI)	C.I (95%)	Pval
rs1805005	rs1270054	0.76	0.72-0.79	$3.04x10^{-11}$	rs1129038	0.66	0.62-0.69	$1.73x10^{-14}$	rs6059655	3.14	3.0-3.28	$4.04x10^{-143}$
rs1805006	NS	NS	NS	NS	NS	NS	NS	NS	NS	NS	NS	NS
rs2228479	NS	NS	NS	NS	NS	NS	NS	NS	rs6059655	3.45	3.22-3.76	$4.41x10^{-57}$
rs34158934	NS	NS	NS	NS	NS	NS	NS	NS	NS	NS	NS	NS
rs11547464	NS	NS	NS	NS	NS	NS	NS	NS	NS	NS	NS	NS
rs1805007	NS	NS	NS	NS	NS	NS	NS	NS	rs6088372	0.81	0.78-0.83	$1.36x10^{-15}$
rs201326893	NS	NS	NS	NS	NS	NS	NS	NS	NS	NS	NS	NS
rs1110400	NS	NS	NS	NS	NS	NS	NS	NS	rs56144975	1.79	1.62-1.96	$7.08x10^{10}$
rs1805008	NS	NS	NS	NS	rs1129038	0.84	0.82-0.86	$1.24x10^{-10}$	NS	NS	NS	NS
rs885479	NS	NS	NS	NS	NS	NS	NS	NS	rs6059655	2.38	2.16-2.65	$1.29x10^{-17}$
rs1805009	NS	NS	NS	NS	NS	NS	NS	NS	rs117119427	0.73	0.70-0.77	$8.91x10^{-11}$
rs368507952	NS	NS	NS	NS	NS	NS	NS	NS	NS	NS	NS	NS

Supplementary Table 4. Blonde Hair GWAS. Blonde hair vs black plus brown GWAS, including analysis following serial conditioning for variants chromosome by chromosome

Chromosome	Locus	Variant	Minor Allele	OR	Z-score	p.value	Position	Annotation	MAF	gene 1	gene 2	PICS
1	SDF4/ TNFRS4	rs75972122	C	1.066	5.98	2.11E-09	1151973	down/upstream	0.151	SDF4	TNFRSF4	6
1	UTS2	rs11582820	T	1.131	7.424	1.14E-13	7950848	Intron	0.05249	UTS2		1
1	TNFRSF9	rs76648881	C	1.248	9.966	2.15E-23	8007595	Intergenic	0.02603	TNFRSF9	PARK7	2
1	SLC45A1	rs147458259	T	1.416	12.39	2.85E-35	8243102	Intergenic	0.01466	ERRFI1	SLC45A1	1
1	SLC45A1	rs77905678	T	1.366	8.525	1.53E-17	8263108	Intergenic	0.008757	SLC45A1	ERRFI1	1
1	PEX14	rs6687430	G	1.045	5.671	1.42E-08	10633245	Intron	0.4588	PEX14		2
1	c1orf127	rs112115136	A	1.119	12.5	7.34E-36	11037434	Intron	0.2198	C1Orf127		21
1	UQCRHL	rs12738340	C	1.09	6.935	4.08E-12	16133396	downstream	0.1021	UQCRHl		65
1	PADI3	rs144080386	T	1.304	6.912	4.77E-12	17597423	missense	0.008287	PADI3		1
1	PADI3	rs11203346	G	1.07	6.695	2.16E-11	17600822	Intron	0.1662	PADI3		31
1	PIGV	rs112535818	G	1.082	5.727	1.02E-08	27122152	Intron	0.08082	PIGV		116
1	PTAFR	rs1629168	A	0.9524	-5.15	3.49E-08	28506065	Intron	0.2768	PTAFR		53
1	NFIA	rs17377218	G	0.9121	-5.866	3.60E-09	61700259	Intron	0.06899	NFIA		30
1	WDR63	rs12034421	A	1.05	6.302	2.93E-10	85528006	UTR 5 prime	0.3877	WDR63		45
1	DSTYK	rs2369633	T	1.213	15.52	1.82E-54	205181062	Intron	0.09145	DSTYK		3
1	KCNH1	rs1338356	C	0.9466	-5.786	7.22E-09	211352625	Intergenic	0.2148	KCNH1	RCOR3	33
1	CDC42BPA	rs11806180	C	0.9537	-5.67	1.43E-08	227503469	Intron	0.3114	CDC42BPA		212
1	FMN2	rs7550088	T	0.9554	-5.598	2.16E-08	240402653	Intron	0.3595	FMN2		9
2	BABAM2	rs4665412	T	0.9562	-5.891	3.85E-09	28585808	Intergenic	0.4775	BABAM2	FOSL2	18
2	FLJ31356 / FOSL2	rs11680860	A	1.119	12.31	8.45E-35	28614304	intron/upstream	0.2214	FOSL2		1
2	FOSL2	rs62139588	A	0.9305	-5.498	3.84E-08	28659534	Intergenic	0.1436	FOSL2	PLB1	1
2	c2orf91	rs13419021	G	0.9114	-11.47	1.85E-30	42167323	Intron	0.3832	C2Orf91		10
2	THNSL2	rs6707137	A	1.129	7.807	5.85E-15	88554351	Intergenic	0.06251	THNSL2	FOXI3	25
2	EN1	rs13035328	G	0.9401	-7.023	2.18E-12	119555976	Intergenic	0.2807	EN1	INSIG2	15
2	TMEM163	rs6739706	A	1.063	7.654	1.95E-14	135407409	Intron	0.389	TMEM163		23
2	MTX2	rs12693099	C	0.9376	-6.398	1.58E-10	177603719	Intergenic	0.4017	MTX2	HNRNPA3	8
2	MYO1B	rs12614848	A	0.9525	-5.71	1.13E-08	192096719	Intergenic	0.3273	MYO1B	STAT4	30
2	FZD7	rs2882325	C	0.947	-6.913	4.75E-12	202838874	Intergenic	0.4605	FZD7	CDK15	3
2	EPHA4	rs10169459	T	1.163	19.71	1.64E-86	222051419	Intron	0.4203	EPHA4		1
2	EPHA4	rs1432262	T	0.8878	-10.24	1.31E-24	222067447	Intergenic	0.4114	EPHA4		3
2	EPHA4	rs17349283	G	1.141	16.98	1.14E-64	222089797	Intergenic	0.4534	EPHA4		4

2	EPHA4	rs16862425	A	0.8775	-7.316	2.56E-13	222141044	Intergenic	0.08397	EPHA4		17
2	PAX3	rs13017777	T	1.056	6.966	3.26E-12	223069625	Intron	0.4985	PAX3		2
2	PAX3	rs12618431	G	0.9243	-6.421	1.35E-10	223110512	Intron	0.1243	PAX3		37
2	PAX3	rs12618491	A	0.9404	-7.2	6.03E-13	223133508	Intron	0.3887	PAX3		1
2	TWIST2	rs9287636	G	1.06	6.971	3.15E-12	239680992	Intergenic	0.3109	TWIST2	ASB1	1
2	HDAC4	rs12185725	G	1.068	6.743	1.55E-11	239949823	Intergenic	0.1947	HDAC4	TWIST2	2
3	CHL1	rs9809528	G	1.043	5.464	4.65E-08	250758	Intron	0.4383	CHL1		3
3	VGLL4	rs2443723	G	0.9388	-5.699	1.21E-08	11662292	Intron	0.1455	VGLL4		19
3	MITF	rs9825958	T	1.059	7.433	1.06E-13	69830674	Intron	0.4156	MITF		68
3	DIRC2	rs9847240	G	0.9152	-10.81	3.21E-27	122526816	Intron	0.3314	DIRC2		7
3	MRAS	rs6782181	G	1.045	5.522	3.36E-08	138105054	Intron	0.3517	MRAS		48
3	ZBTB38	rs4683605	A	1.131	16.06	4.73E-58	141094769	Intron	0.4464	ZBTB38		13
3	ATP1B3	rs3804772	A	1.105	8.929	4.30E-19	141634056	Intron	0.1253	ATP1B3		54
3	MBNL1	rs325712	C	1.049	6.036	1.58E-09	151900673	Intergenic	0.3657	MBNL1	SUCNR1	58
4	AREG	rs1874202	G	0.9256	-9.813	9.88E-23	75328479	Intergenic	0.4222	AREG	BTC	7
4	FRAS1	rs1268789	T	0.9235	-9.517	1.78E-21	79280693	Intron	0.326	FRAS1		17
4	FGF5	rs1458046	A	1.072	8.854	8.44E-19	81199966	Intron	0.4044	FGF5		7
4	c4orf22	rs62302224	G	1.064	7.566	3.84E-14	81852606	intron	0.3439	C4Orf22		1
4	ARHGAP24	rs1026872	T	0.9436	-7.395	1.42E-13	86601631	Intron	0.4881	ARHGAP24		4
4	TET2	rs2522490	A	1.056	6.783	1.75E-11	105778330	Intergenic	0.37	TET2	CXXC4	7
4	LEF1	rs116711774	A	0.7466	-5.977	2.01E-09	109020802	Intron	0.00822	LEF1		23
4	LEF1	rs922168	T	0.9313	-7.913	2.52E-15	109057404	Intron	0.2625	LEF1		7
4	RPL34	rs219493	T	0.907	-9.234	2.61E-20	109350880	intergenic	0.1698	RPL34	LEF1	1
4	RPL34-AS1	rs11731416	C	1.074	9.229	2.74E-20	109478108	upstream	0.4909	RPL34	LEF1	28
4	NR3C2	rs4407483	C	1.083	6.97	3.16E-12	149856841	Intergenic	0.1314	NR3C2	IQCM	7
5	ADAMTS12	5:33904113_G	A	17.39	18.66	1.12E-77	33904113	Intergenic	0.000305	ADAMTS12	RXFP3	1
5	SLC45A2	rs16891982	C	0.304	-28.71	3.10E-181	33951693	missense	0.0228	SLC45A2		1
5	SLC45A2	rs116887602	C	3.921	6.61	3.84E-11	33963850	stop gained	0.000159	SLC45A2		1
5	SLC45A2	rs201259497	T	4.585	9.704	2.91E-22	33964091	missense	0.000268	SLC45A2		1
5	SLC45A2	rs13289	C	1.087	10.52	6.76E-26	33986409	upstream	0.3727	SLC45A2		1
5	NDUFS4	rs62370277	T	0.9004	-8.468	2.50E-17	53067320	Intergenic	0.1332	NDUFS4	ARL15	5
5	LINC02105	rs6875907	C	0.909	-12.41	2.43E-35	53112624	Intron	0.4706	NDUFS4	ARL15	8
5	LOC105378966	rs17248377	A	1.073	6.72	1.82E-11	53116123	upstream	0.2398	NDUFS4	ARL15	2

5	MAP3K1	rs61055995	T	0.9346	-6.338	2.33E-10	56019064	Intergenic	0.1628	MAP3K1	c5orf67	12
5	PLK2	rs6868805	C	0.9481	-6.249	4.13E-10	57430239	Intergenic	0.3008	PLK2	ACTBL2	11
5	ZFYVE16	rs259035	G	0.9237	-5.669	1.44E-08	79695370	Intergenic	0.08862	ZFYVE16	SPZ1	66
5	ADGRV1	rs6860111	G	0.8897	-14.6	2.81E-48	90263581	Intron	0.3706	ADGRV1		21
5	NSG2	rs2964049	G	0.9519	-6.099	1.07E-09	173830622	Intergenic	0.3715	NSG2	MSX2	13
5	MSX2	rs4242182	T	1.095	7.259	3.91E-13	174156168	missense	0.09754	MSX2		4
6	IRF4	rs2671427	T	1.07	8.38	5.28E-17	385735	Intergenic	0.3731	IRF4	DUSP22	1
6	IRF4	rs74758148	A	1.128	6.374	1.84E-10	386933	Intergenic	0.04255	IRF4	DUSP22	1
6	IRF4	rs3778607	A	1.08	7.022	2.19E-12	403799	Intron	0.4555	IRF4		1
6	IRF4	rs9392504	G	0.8526	-8.82	1.15E-18	412802	Intergenic	0.4574	IRF4	EXOC2	2
6	IRF4	rs4246064	C	0.9219	-8.627	6.28E-18	421196	Intergenic	0.3884	IRF4	EXOC2	3
6	IRF4	rs62389423	A	0.3866	-68.4	2.25E-308	421281	Intergenic	0.172	IRF4	EXOC2	1
6	IRF4	rs143615986	A	0.5669	-18.44	6.01E-76	433066	Intergenic	0.02282	IRF4	EXOC2	1
6	EXOC2	rs6918152	A	1.137	16.25	2.31E-59	542159	intron	0.363	EXOC2		4
6	CDKAL1	rs78287738	T	0.946	-6.595	4.25E-11	20550066	Intron	0.3175	CDKAL1		20
6	MSH5	rs17207524	T	1.09	5.539	3.04E-08	31726850	Intron	0.06447	MSH5		16
6	CLIC5	rs9349337	A	0.9483	-6.91	6.02E-10	45901916	Intron	0.3026	CLIC5		22
6	SGK1	rs4896038	C	1.053	5.548	1.88E-08	134609291	Intron	0.2241	SGK1		1
6	LOC100507477/LOC	rs1416288	A	1.051	5.523	3.34E-08	140313718	Intron	0.2425	coding gene desert		22
6	AKAP12	rs4869723	T	1.058	7.232	4.75E-13	151579432	Intron	0.439	AKAP12		4
6	EZR	rs3212308	C	0.8869	-15.59	8.90E-55	159191788	Intron	0.4879	EZR		20
6	EZR	rs73013664	T	1.134	10.92	9.69E-26	159260987	Intergenic	0.18	EZR	RSPH3	8
7	ETV1	rs3213661	A	1.0588	6.68	2.38E-11	14026357	Intron	0.2852	ETV1		6
7	JAZF1	rs864745	T	1.071	8.994	2.38E-19	28180556	Intron	0.4946	JAZF1		9
7	CREB5	rs28531809	C	0.9572	-5.509	3.62E-08	28803911	Intron	0.3807	CREB5		7
7	FZD1	rs12667582	T	0.8443	-11.36	6.43E-30	90848218	intergenic	0.08033	CDK14	FZD1	1
7	MTERF1	rs2710956	G	1.046	5.814	6.09E-09	91190424	Intergenic	0.4505	MTERF1	FZD1	13
7	EPHB4	rs314349	G	0.9465	-6.838	8.01E-12	100401825	Intron	0.3667	EPHB4		3
7	SLC12A9	rs12535629	T	1.07	7.959	1.74E-15	100451732	intron	0.28	SLC12A9		8
7	SLC12A9	rs80308281	C	1.696	12.24	1.85E-34	100457578	missense	0.005442	SLC12A9		1
7	ATXN7L1	rs2529369	A	0.9365	-7.621	2.52E-14	105416560	Intron	0.2836	ATXN7L1		1
7	LINC-PINT	rs10954300	G	0.9317	-8.037	9.19E-16	130761235	Intron	0.2622	KLF14	MKLN1	2
8	PEBP4	rs7845221	T	1.064	7.067	1.58E-12	22599421	intron	0.2546	PEBP4		1

8	TACC1	rs57128498	A	1.074	6.828	8.60E-12	38568215	Intergenic	0.1557	TACC1	c8orf86	1
8	AP3M2	rs113060680	T	1.078	6.403	1.52E-10	41987884	Intergenic	0.1172	AP3M2	KAT6A	20
8	SNX16	rs2600605	A	1.087	9.78	1.37E-22	82720760	intron	0.2622	SNX16		61
8	RSPO2	rs117954051	C	1.262	7.442	9.94E-14	109067613	intron	0.01248	RSPO2		5
8	RSPO2	rs377057	T	0.9461	-6.194	5.86E-10	109142307	Intergenic	0.2539	RSPO2	EIF3E	73
9	DOCK8	rs520015	G	0.9204	-10.74	6.56E-27	211762	Intron	0.4883	DOCK8		3
9	ERMP1	rs2093657	C	1.055	6.549	5.80E-11	5783498	Intron	0.3377	ERMP1		64
9	KIAA2026	rs10815302	A	0.7342	-6.204	5.49E-10	5887074	Intron	0.007996	KIAA2026		72
9	TYRP1	rs13288130	T	0.8611	-18.64	1.39E-77	12712578	Intron	0.3706	TYRP1	LURAP1L	12
9	BNC2	rs10962599	C	0.9144	-9.656	4.62E-22	16795286	Intron	0.2365	BNC2		7
9	BNC2	rs12350739	G	0.9093	-11.91	1.04E-32	16885017	Intergenic	0.3871	BNC2	CNTLN	6
9	FOXE1	rs7873389	C	0.9308	-8.957	3.32E-19	100609230	Intergenic	0.3885	FOXE1	XPA	34
9	TMEM38B	rs1484372	G	1.064	6.66	2.74E-11	109010799	Intergenic	0.2159	TMEM38B	ZNF462	5
9	LHX2	rs917783	C	1.138	16.92	3.10E-64	126790607	intron	0.4546	LHX2		13
9	LHX2	rs10114314	T	0.8761	-9.317	1.19E-20	126808788	Intergenic	0.1074	LHX2	NEK6	8
9	LHX2	rs12344562	C	1.199	18.74	2.50E-78	126811296	Intergenic	0.184	LHX2	NEK6	3
9	NEK6	rs72759273	G	1.076	7.545	4.53E-14	126973873	Intergenic	0.1917	LHX2	NEK6	3
9	NEK6	rs10818930	T	0.916	-8.263	1.42E-16	126991184	intergenic	0.1826	NEK6	LHX2	3
10	DDIT4	rs7075993	G	1.052	6.432	1.26E-10	74054590	Intergenic	0.3548	DDIT4	DNAJB2	35
10	ZMIZ1	rs703978	C	0.9253	-9.99	1.68E-23	80944147	Intron	0.4173	ZMIZ1		5
11	PPFIBP2	rs11041426	G	0.8957	-13.68	1.42E-42	7543519	Intron	0.3818	PPFIBP2		1
11	SOX6	rs1531903	C	0.9196	-7.838	4.59E-15	15668826	Intergenic	0.1665	SOX6	INSC	2
11	SOX6	rs7109376	A	1.068	7.706	1.30E-14	16372431	Intron	0.2792	SOX6		5
11	SOX6	rs7943712	A	0.9376	-6.398	1.58E-10	16543863	Intron	0.195	SOX6		69
11	LGR4	rs58604758	A	1.12	7.441	1.00E-13	27420145	Intron	0.06516	LGR4		23
11	CD82	rs357925	A	1.064	6.673	2.51E-11	44496552	intergenic	0.2254	CD82	ALX4	20
11	MYRF	rs61896141	C	1.089	8.8	1.37E-18	61556039	downstream	0.1893	MYRF		3
11	AHNAK	rs10897275	A	1.085	9.842	7.42E-23	62203865	Intron	0.3153	AHNAK		2
11	OVOL1	rs56019505	G	1.087	7.589	3.23E-14	65561805	Intron	0.14	OVOL1		15
11	TPCN2	rs72917317	G	1.545	39.23	2.25E-308	68817441	Intron	0.1064	TPCN2		2
11	MIR3164/TYR	rs34510004	A	3.434	16.17	8.15E-59	68848916	missense/upstream	0.001323	TPCN2		1
11	TPCN2	rs3829241	A	1.269	29.25	4.73E-188	68855363	missense	0.4065	TPCN2		1
11	CCND1	rs12806763	C	0.9406	-7.248	4.24E-13	69358817	Intergenic	0.3304	CCND1	MYEOV	39

11	GAB2	rs2292572	T	0.8945	-10.22	1.62E-24	78052864	Intron	0.1587	GAB2		3
11	GRM5	rs148065054	T	1.214	8.331	7.99E-17	88552633	Intron	0.02513	GRM5		1
11	TYR	rs1042602	A	1.325	30.21	1.75E-200	88911696	missense	0.3674	TYR		1
11	TYR	rs1393350	A	1.16	17.86	2.64E-71	89011046	Intron	0.289	TYR		1
12	CCND2	rs3764032	C	0.899	-517	3.55E-08	4317563	Intergenic	0.04596	CCND2	PARP11	3
12	SLC38A2	rs4768698	A	1.048	6.005	1.91E-09	46749088	intergenic	0.3929	SLC38A2	SLC38A1	66
12	KRT86/KRT7	rs139727704	G	1.414	7.017	2.26E-12	52648158	Intron/downstrean	0.004688	KRT86	KRT7	1
12	HOXC13	rs535209331	CTTA	0.835	-5.863	4.55E-09	54332733	inframe insertion	0.01909	HOXC13		1
12	TMTC3	rs7487365	C	1.15	6.38	1.76E-10	88674623	Intergenic	0.06804	TMTC3	KITLG	4
12	TMTC3	rs2216153	G	0.8409	-19.53	6.05E-85	88681977	Intergenic	0.2792	TMTC3	KITLG	1
12	TMTC3	rs7306001	A	1.125	6.844	7.72E-12	88736601	Intergenic	0.07106	TMTC3	KITLG	28
12	KITLG	rs35618688	A	1.113	5.704	1.17E-08	88940157	Intron	0.06058	KITLG		6
12	KITLG	rs1907703	C	0.8907	-10.23	1.50E-24	88955642	Intron	0.2144	KITLG		29
12	KITLG	rs12821256	C	1.659	48.66	2.25E-308	89328335	Intergenic	0.1194	KITLG	DUSP6	1
12	KITLG	rs12298351	T	0.8557	-6.682	2.35E-11	89340112	Intergenic	0.03415	KITLG	DUSP6	4
12	RPL6	rs11066284	A	1.058	5.52	3.48E-08	112842275	Intergenic	0.1713	RPL6	HECTD4	223
12	MED13L	rs61939692	A	1.073	5.867	4.40E-09	116535976	Intron	0.1124	MED13L		19
12	MAP1LC3B2	rs11068059	C	1.052	6.377	1.81E-10	116967670	Intergenic	0.3595	MAP1LC3B2	MED13L	16
13	FREM2	rs9603422	T	0.9235	-6.466	1.01E-10	39343822	missense	0.1154	FREM2		1
13	SLAIN1	rs750192	A	0.8151	-25.63	7.05E-145	78390743	Intergenic	0.3924	SLAIN1	EDNRB	29
13	DCT	rs6492711	T	1.175	20.37	2.86E-92	95196559	Intron	0.343	DCT		4
14	BMP4	rs210381	G	1.044	5.501	3.77E-08	54107791	Intergenic	0.4268	BMP4	DDHD1	12
14	PPM1A	rs1887103	G	1.059	7.124	1.05E-12	60743219	Intron	0.3564	PPM1A		38
14	SYNE2	rs10873172	G	1.068	7.804	5.98E-15	64390030	Intron	0.2816	SYNE2		47
14	RAD51B	rs11158717	G	0.9417	-6.041	1.54E-09	68514276	Intron	0.1956	RAD51B		111
14	ZFP36L1	rs72731537	T	0.9314	-6.867	6.55E-12	69237925	Intergenic	0.1767	ZFP36L1	RAD51B	21
14	SLC24A4	rs75433889	T	1.109	7.144	9.06E-13	92726294	Intergenic	0.06872	SLC24A4	CPSF2	2
14	LOC105370627	rs941799	T	1.662	65.49	2.25E-308	92776825	Intergenic	0.4533	SLC24A4	CPSF2	4
14	SLC24A4	rs4904886	A	1.052	6.057	1.39E-09	92844370	Intron	0.3211	SLC24A4		4
14	SLC24A4	rs17783630	A	1.062	7.753	8.98E-15	92955385	Intron	0.441	SLC24A4		2
14	MARK3	rs55859054	A	0.9512	-6.144	9.71E-10	103953666	Intron	0.3529	MARK3		76
15	OCA2	rs139029488	C	1.52	9.203	3.47E-20	27946226	Intergenic	0.006794	OCA2	GABRG3	1
15	OCA2	rs924318	A	1.097	11.15	7.38E-29	28093434	Intron	0.3417	OCA2		11

15	OCA2	rs121918167	A	10.35	12.06	1.83E-33	28116316	missense	0.000197	OCA2		1
15	OCA2	rs72625132	C	1.225	14.77	2.36E-49	28213924	Intron	0.1125	OCA2		9
15	OCA2	rs121918170	C	6.071	22.05	9.61E-108	28228529	missense	0.001026	OCA2		1
15	OCA2	rs74653330	T	9.295	19.46	2.73E-84	28228553	missense	0.000652	OCA2		1
15	OCA2	rs121918166	T	5.937	54.27	2.25E-308	28230247	missense	0.009077	OCA2		1
15	OCA2	rs1800407	T	1.399	19.11	2.20E-81	28230318	missense	0.08538	OCA2		1
15	OCA2	rs4778224	A	0.8468	-11.24	2.46E-29	28241020	intron	0.1614	OCA2		1
15	OCA2	rs116978932	A	1.139	5.762	8.32E-09	28324912	Intron	0.05458	OCA2		9
15	HERC2	rs12913832	A	0.2132	-101.9	2.25E-308	28365618	Intron	0.2135	HERC2		1
15	HERC2	rs75165924	T	1.41	9.94	2.78E-21	28380258	Intron	0.04666	HERC2		67
15	HERC2	rs77572354	G	1.277	6.837	8.08E-12	28560722	Intron	0.08222	HERC2		13
15	IL6	rs67093094	T	1.151	14.85	7.36E-50	81530848	Intron	0.2049	IL6		41
15	BNC1	rs8033380	C	1.07	7.74	9.96E-15	83957217	Intergenic	0.2779	BNC1	SH3GL3	16
16	CDK10	rs116927526	T	2.283	14.79	1.78E-49	89743627	Intergenic	0.004391	CDK10	SPATA33	1
16	SPATA2L	rs35432452	G	1.125	9.843	7.45E-23	89765046	Intron	0.1462	SPATA2L		1
16	TCF25	rs182948919	T	1.507	12.03	2.39E-33	89938244	upstream	0.01219	TCF25		18
16	TCF25	rs9939914	C	1.14	12.5	7.26E-36	89939929	upstream	0.2504	TCF25		1
16	MC1R	rs1805005	T	1.314	23.82	2.22E-125	89985844	missense	0.122	MC1R		1
16	MC1R	rs1805006	A	1.816	18.8	6.95E-79	89985918	missense	0.01312	MC1R		1
16	MC1R	rs11547464	A	1.897	14.9	3.30E-50	89986091	missense	0.007148	MC1R		1
16	MC1R	rs1805007	T	1.744	45.42	2.25E-308	89986117	missense	0.1048	MC1R		1
16	MC1R	rs1805008	T	1.77	45.05	2.25E-308	89986144	missense	0.08918	MC1R		1
16	MC1R	rs885479	A	1.165	5.867	4.44E-08	89986154	missense	0.04791	MC1R		
16	MC1R	rs555179612	TC	2.465	9.76	1.67E-22	89986202	frameshift	0.001706	MC1R		1
16	MC1R	rs200000734	T	2.59	6.231	4.62E-10	89986303	missense	0.000613	MC1R		1
16	AFG3L1P	rs575866787	A	1.782	22.54	1.87E-112	90052987	Intron	0.02579	AFG3L1P		1
16	DBNDD1	rs77733403	C	1.087	5.46	4.75E-08	90080723	Intron	0.1742	DBNDD1		1
17	WIPF2	rs62065255	C	1.052	5.55	2.85E-08	38409081	Intron	0.2504	WIPF2		2
17	KRT33A	rs140814701	A	0.893	-6.533	6.45E-11	39491979	Intergenic	0.06072	KRT33A	KRTAP17-	2
17	SP6	rs72833470	G	1.143	15.9	6.72E-57	45950721	intron	0.2774	SP6		3
17	SP2	rs16949418	T	0.927	-8.046	8.57E-16	45991240	Intron	0.3028	SP6		7
17	DLX4	rs9303554	T	0.9488	-6.63	3.36E-11	48008683	Intergenic	0.4596	DLX4	TAC4	1
17	DLX4	rs55788912	A	0.911	-10.54	5.75E-26	48022018	Intergenic	0.2725	DLX4	TAC4	1

17	AKAP1	rs17833789	A	1.069	8.486	2.13E-19	55230628	Intergenic	0.4464	AKAP1	MSI2	3
17	AXIN2	rs7406690	G	0.9158	-5.67	1.43E-08	63518525	Intergenic	0.07507	AXIN2	RGS9	3
17	PDE6G	rs35763415	T	1.072	8.873	7.15E-19	79622370	intron/upstream	0.4119	PDE6G		1
17	NOTUM	rs35406919	G	0.9354	-8.535	1.41E-17	79908566	Intergenic	0.4664	NOTUM	PYCR1	13
17	TBCD	rs79316200	T	1.082	6.502	7.92E-11	80893588	Intron	0.1136	TBCD		3
19	TMEM91	rs12602	T	1.055	6.838	8.00E-12	41889748	Intron	0.3821	TMEM91		74
20	RALY	rs6059655	A	1.354	25.98	8.05E-149	32665748	Intron	0.1059	RALY		1
20	BCAS1	rs55901013	T	1.214	11.5	1.35E-30	52642793	Intron	0.04725	BCAS1		8
20	TFAP2C	rs6127868	A	1.092	8.364	6.08E-17	55409093	Intergenic	0.1491	TFAP2C	BMP7	3
20	ZNF831	rs1036464	A	0.9402	-7.26	3.86E-13	57841686	Intergenic	0.2936	ZNF831	EDN3	19
21	MRPL39	rs2829786	G	0.9267	-5.975	2.30E-09	26878642	Intergenic	0.1058	MRPL39		47
21	LINC00322	rs73220980	A	1.142	5.925	3.13E-09	44752768	upstream	0.02766	SIK1	CRYAA	1
21	SIK1	rs672948	A	1.063	7.922	2.34E-15	44793448	Intergenic	0.4145	SIK1	CRYAA	3
22	SYNGR1	rs9611155	T	0.9533	-5.701	1.19E-08	39739187	Intergenic	0.3006	SYNGR1	RPL3	3
22	KIAA0930	rs2294196	T	0.9552	-5.987	2.14E-09	45630662	intron	0.4673	KIAA0930		15
22	ATXN10	rs136047	A	0.8876	-5.635	1.75E-08	46264381	Intergenic	0.03693	ATXN10	WNT7B	7
22	PLXNB2	rs79966207	C	1.063	6.233	4.57E-10	50722408	missense	0.1776	PLXNB2		1

Supplementary Table 5 *MC1R* Variants and Hair Colour. The number of *MC1R* coding variants, plus rs3212379, found in individuals with each hair colour. Individuals with 3 variants are omitted, as are those who gave hair colour as "other" or "no answer". Percentages are hair colour fractions with 0, 1 or 2 variants

	number of MC1R variants					
	0		1		2	
Red	100	0.10%	989	0.60%	14471	15%
Blonde	5975	8%	19275	11.50%	13971	15%
Light brown	29561	39%	72808	43%	38636	41%
Dark brown	35477	47%	67223	40%	25044	27%
Black	4749	6%	7636	4.50%	2126	2%
Total	75862		167931		94248	

Supplementary Table 6. Comparison to Hysi et al. Lead SNPs from Hysi et al, P value from their work and the best model in our study and the OR from the selected model.
"This study P" is the logistic P-value from this study. 1kb – 500kb, presence of significant associations in this study within the given genomic interval from the Hysi et al significant association.
1=significant association present, 0=not present. NA=variant from Hysi et al not analysed in this study

CHR	SNP	BP	A1	P.Hysi	MODEL	OR	this study P	1Kb	5Kb	10Kb	50Kb	100Kb	500Kb
1	rs79361800	1177211	C	3.05E-11	blonde	1.065	4.41E-07	0	1	1	1	1	1
1	rs6689838	11035264	A	1.29E-25	blonde	1.119	9.14E-36	1	1	1	1	1	1
1	rs72646785	17603472	T	4.54E-15	blonde	1.065	4.07E-10	1	1	1	1	1	1
1	rs3856254	41883722	A	1.54E-08	blonde	1.024	0.002302	0	0	0	0	0	0
1	rs17377232	61702673	C	5.17E-10	blonde	0.9083	6.88E-10	1	1	1	1	1	1
1	rs12134456	155722506	G	1.90E-10	blonde	0.973	0.0006148	0	0	0	0	0	0
1	rs1323292	192541021	G	2.61E-13	blonde	1.043	2.14E-05	0	0	0	0	0	0
1	rs2369633	205181062	T	6.08E-61	blonde	1.213	2.75E-54	1	1	1	1	1	1
1	rs6698338	211213706	C	7.61E-09	blonde	0.9667	1.49E-05	0	0	0	0	0	1
1	rs1338349	211289337	A	5.51E-11	blonde	0.9499	5.61E-07	0	0	0	1	1	1
1	rs11806180	227503469	C	2.11E-13	blonde	0.9534	8.24E-09	1	1	1	1	1	1
1	rs12749578	232625553	A	1.34E-08	blonde	0.9611	6.19E-06	0	0	0	0	0	0
2	rs4952542	42148519	T	2.05E-33	blonde	0.9127	1.30E-30	1	1	1	1	1	1
2	rs73952210	88588510	C	2.65E-15	blonde	1.086	1.73E-12	1	1	1	1	1	1
2	rs6728095	135376072	T	7.62E-12	blonde	1.061	1.99E-14	1	1	1	1	1	1
2	rs726357	202841043	G	4.38E-16	blonde	1.053	1.30E-11	1	1	1	1	1	1
2	rs78992409	214064601	C	4.95E-10	blonde	0.9611	0.005831	0	0	0	0	0	0
2	rs10169459	222051419	T	1.00E-100	blonde	1.163	1.65E-86	1	1	1	1	1	1
2	rs11684254	239695893	G	9.49E-19	blonde	1.06	4.41E-13	1	1	1	1	1	1
3	rs13082190	251422	C	2.10E-09	blonde	0.9688	6.91E-05	0	0	0	0	0	0
3	rs2574715	11663025	A	1.77E-14	blonde	0.9404	8.33E-08	1	1	1	1	1	1
3	rs9823839	69880602	C	8.75E-14	blonde	1.057	4.61E-13	1	1	1	1	1	1
3	rs9821691	72396902	T	3.05E-09	blonde	0.9577	2.60E-08	1	1	1	1	1	1
3	rs586936	73862616	A	1.45E-10	blonde	0.9634	2.02E-06	0	0	0	0	0	0
3	rs9847240	122526816	G	1.19E-26	blonde	0.9155	5.45E-27	1	1	1	1	1	1
3	rs6440008	141154542	C	5.35E-54	blonde	1.125	1.98E-51	1	1	1	1	1	1
4	rs2117599	54465287	A	7.63E-10	blonde	0.9538	1.24E-08	1	1	1	1	1	1
4	rs1874202	75328479	G	2.50E-19	blonde	0.9256	9.88E-23	1	1	1	1	1	1
4	rs7681907	81205868	A	1.88E-24	blonde	1.071	3.34E-19	1	1	1	1	1	1
4	rs1026873	86601669	T	1.97E-11	blonde	0.9467	9.39E-13	1	1	1	1	1	1
4	rs72656294	86684641	G	1.85E-08	blonde	0.9745	0.01322	0	0	0	0	1	1
4	rs9998015	105816898	C	1.79E-20	blonde	1.054	2.18E-11	1	1	1	1	1	1
4	rs1436502	109482494	C	8.13E-21	blonde	1.074	5.97E-21	1	1	1	1	1	1
4	rs7672648	109482831	C	3.24E-20	blonde	1.073	1.61E-20	1	1	1	1	1	1
4	rs72737816	149745038	A	4.05E-08	blonde	1.08	4.04E-12	1	1	1	1	1	1
5	rs16891982	33951693	C	1.00E-100	brown	0.3529	0	1	1	1	1	1	1
5	rs1504212	53119955	C	3.80E-36	blonde	0.9101	1.96E-34	1	1	1	1	1	1
5	rs271205	53192812	A	3.74E-11	blonde	0.9216	1.12E-19	1	1	1	1	1	1
5	rs1835873	57135050	C	5.80E-09	blonde	0.9681	2.74E-05	0	0	0	0	0	1
5	rs7700279	59016897	G	4.22E-08	brown	1.049	0.0002503	0	0	0	0	0	0
5	rs259035	79695370	G	1.47E-11	blonde	0.9232	6.45E-09	1	1	1	1	1	1
5	rs1995774	90276193	A	2.34E-49	blonde	0.89	3.52E-48	1	1	1	1	1	1
5	rs10519488	116231256	G	1.86E-11	blonde	0.9196	6.02E-06	0	0	0	0	0	0
5	rs10051152	133849987	T	3.01E-10	blonde	1.036	6.27E-05	0	0	0	0	0	0
5	rs12054866	133894781	T	3.62E-12	blonde	1.057	8.99E-07	0	0	0	0	0	0
5	rs4242182	174156168	T	1.72E-13	blonde	1.094	8.04E-13	1	1	1	1	1	1
6	rs12208597	20562740	C	3.54E-11	blonde	0.9551	5.40E-09	1	1	1	1	1	1
6	rs16886790	22710500	C	7.94E-09	blonde	1.033	0.001336	0	0	0	0	0	0
6	rs2233981	31079578	A	7.67E-10	blonde	0.949	5.20E-05	0	0	0	0	0	0
6	rs9463733	51722693	G	2.67E-08	red	0.8683	2.83E-28	1	1	1	1	1	1
6	rs9492790	131368493	C	1.17E-09	brown	0.9439	4.59E-05	0	0	0	0	0	0
6	rs374522	134648414	T	1.10E-10	blonde	1.031	0.0001304	0	0	0	1	1	1
6	rs4869723	151579432	T	5.86E-17	blonde	1.056	1.66E-12	1	1	1	1	1	1
6	rs9347258	159233043	C	9.67E-78	blonde	0.8903	1.97E-52	1	1	1	1	1	1
7	rs3801108	14025411	T	1.72E-08	blonde	1.05	2.06E-10	1	1	1	1	1	1
7	rs849134	28196222	A	2.37E-15	blonde	1.068	7.63E-18	1	1	1	1	1	1
7	rs34764931	41889049	G	7.23E-09	blonde	1.033	2.41E-05	0	0	0	0	0	0
7	rs12702237	46804207	T	9.65E-17	blonde	0.9566	6.35E-07	0	0	0	0	0	0
7	rs12667582	90848218	T	1.09E-26	blonde	0.8446	8.37E-30	1	1	1	1	1	1
7	rs2075756	100466441	A	2.64E-20	blonde	1.079	1.68E-19	1	1	1	1	1	1
7	rs2529369	105416560	A	2.15E-16	blonde	0.9367	2.29E-14	1	1	1	1	1	1
7	rs12706959	130743676	T	8.51E-27	blonde	0.9343	1.65E-12	1	1	1	1	1	1
8	rs17676443	22601427	T	1.01E-09	blonde	0.9532	2.02E-09	1	1	1	1	1	1
8	rs2595041	29852712	C	6.57E-10	blonde	1.028	0.0005332	0	0	0	0	0	0
8	rs57128498	38568215	A	1.85E-14	blonde	1.073	9.65E-12	1	1	1	1	1	1

8	rs774470	82724326	C	7.94E-38	blonde	1.087	2.23E-22	1	1	1	1	1	1
8	rs424012	109154652	A	2.63E-15	blonde	1.041	1.61E-07	1	1	1	1	1	1
9	rs478882	205964	G	6.07E-21	blonde	0.9221	2.01E-26	1	1	1	1	1	1
9	rs2233173	5890270	T	4.84E-10	blonde	0.8958	1.15E-07	0	1	1	1	1	1
9	rs10960765	12730119	T	4.57E-67	blonde	0.8656	4.62E-72	1	1	1	1	1	1
9	rs12350739	16885017	G	2.29E-44	blonde	0.9101	2.16E-32	1	1	1	1	1	1
9	rs9632885	22072638	A	6.16E-09	blonde	0.9721	0.0002225	0	0	0	0	0	0
9	rs3021523	100616583	T	6.64E-25	blonde	0.9294	5.41E-17	1	1	1	1	1	1
9	rs10739220	109054417	C	7.47E-14	blonde	1.061	6.24E-11	1	1	1	1	1	1
9	rs58979150	126808006	T	8.06E-92	blonde	0.8017	5.66E-63	1	1	1	1	1	1
10	rs7074233	74050218	A	2.22E-12	blonde	1.049	2.21E-09	1	1	1	1	1	1
10	rs703978	80944147	C	2.43E-37	blonde	0.9253	1.68E-23	1	1	1	1	1	1
10	rs7088364	112775661	A	3.22E-08	brown	1.061	5.01E-05	0	0	0	0	0	0
11	rs11041426	7543519	G	3.41E-37	blonde	0.896	2.49E-43	1	1	1	1	1	1
11	rs1531903	15668826	C	3.93E-15	blonde	0.9241	4.50E-14	1	1	1	1	1	1
11	rs12419588	18301130	A	5.33E-10	blonde	0.9544	5.66E-08	1	1	1	1	1	1
11	rs66716358	44330610	T	6.46E-12	blonde	1.041	1.03E-07	1	1	1	1	1	1
11	rs9645690	62206288	T	4.34E-20	blonde	1.084	1.85E-23	1	1	1	1	1	1
11	rs72917317	68817441	G	1.00E-100	blonde	1.545	0	1	1	1	1	1	1
11	rs1042602	88911696	A	1.00E-100	blonde	1.148	5.44E-70	1	1	1	1	1	1
12	rs11834692	4317819	G	3.99E-13	brown	0.8553	5.73E-09	1	1	1	1	1	1
12	rs9971729	23979791	A	2.29E-17	blonde	1.042	1.18E-07	0	0	0	0	0	0
12	rs10875910	49402393	C	4.48E-09	blonde	0.9676	3.59E-05	0	0	0	0	0	0
12	rs7974210	65186838	C	6.71E-12	blonde	0.9675	1.94E-05	0	0	0	0	0	0
12	rs12821256	89328335	C	1.00E-100	blonde	1.659	0	1	1	1	1	1	1
13	rs9603422	39343822	T	1.48E-11	blonde	0.9249	1.91E-10	1	1	1	1	1	1
13	rs1279403	78391757	C	1.00E-100	blonde	0.8151	6.87E-144	1	1	1	1	1	1
13	rs717769	95162362	G	5.57E-68	blonde	1.172	7.94E-89	1	1	1	1	1	1
13	rs2025905	111184256	G	9.34E-09	brown	0.9613	0.002365	0	0	0	0	0	0
14	rs10873172	64390030	G	2.71E-20	blonde	1.068	4.13E-15	1	1	1	1	1	1
14	rs10139386	68550540	G	6.01E-09	blonde	0.9413	4.74E-09	1	1	1	1	1	1
14	rs56232028	69247948	A	7.16E-13	blonde	0.9316	3.92E-12	1	1	1	1	1	1
14	rs17184180	92780387	A	1.00E-100	blonde	1.662	0	1	1	1	1	1	1
15	rs12913832	28365618	A	1.00E-100	blonde	0.2132	0	1	1	1	1	1	1
15	rs1426654	48426484	G	2.17E-19	blonde	0.492	2.98E-05	0	0	0	0	0	0
15	rs61219147	81533762	G	1.15E-39	blonde	1.147	1.93E-50	1	1	1	1	1	1
16	rs1805007	89986117	T	1.00E-100	red	10.98	0	1	1	1	1	1	1
17	rs117612447	39551099	T	6.17E-15	blonde	0.8558	7.64E-11	1	1	1	1	1	1
17	rs72833466	45948952	A	4.14E-90	blonde	1.142	2.43E-56	1	1	1	1	1	1
17	rs62060349	55231168	C	5.98E-19	blonde	1.067	3.52E-17	1	1	1	1	1	1
18	rs9954676	46353585	T	3.46E-10	blonde	1.039	0.0003168	0	0	0	0	0	0
19	rs11085749	10961273	A	2.96E-09	brown	0.9494	2.40E-05	0	0	0	0	0	0
20	rs6059655	32665748	A	1.00E-100	red	1.879	0	1	1	1	1	1	1
20	rs73132911	52661068	C	7.08E-27	blonde	1.21	1.02E-29	1	1	1	1	1	1
20	rs4811760	55411807	T	9.45E-18	blonde	1.089	3.10E-16	1	1	1	1	1	1
20	rs6128521	57842217	C	4.33E-32	blonde	0.9396	1.60E-13	1	1	1	1	1	1
21	rs60841620	26834111	G	9.90E-14	blonde	0.9296	8.58E-09	1	1	1	1	1	1
21	rs478075	44792798	C	5.94E-27	blonde	0.9438	4.91E-14	1	1	1	1	1	1
22	rs136402	41598933	A	5.67E-11	blonde	0.9691	0.0002144	0	0	0	0	0	0
1	rs80293268	8207579	C	1.00E-100	NA	NA	NA	0	0	0	1	1	1
2	rs71443018	28613302	C	2.15E-65	NA	NA	NA	0	1	1	1	1	1
2	rs13028186	70393127	A	4.47E-08	NA	NA	NA	0	0	0	0	0	0
3	rs67001479	11661526	D	9.06E-11	NA	NA	NA	1	1	1	1	1	1
4	rs10015223	4388874	C	1.38E-09	NA	NA	NA	0	0	0	0	0	0
4	rs72661730	81661426	A	1.87E-39	NA	NA	NA	0	0	0	0	0	1
4	rs142402773	105808536	T	4.64E-12	NA	NA	NA	0	0	1	1	1	1
6	rs12203592	396321	T	1.00E-100	NA	NA	NA	1	1	1	1	1	1
6	rs35735653	45870999	D	2.87E-09	NA	NA	NA	0	0	0	1	1	1
6	rs11280053	113314886	D	2.42E-14	NA	NA	NA	0	0	0	0	0	0
7	rs5882681	18869552	D	5.04E-09	NA	NA	NA	0	0	0	0	0	0
7	rs62471615	130746955	T	2.53E-34	NA	NA	NA	1	1	1	1	1	1
8	rs72110873	42044628	D	2.61E-08	NA	NA	NA	1	1	1	1	1	1
9	rs10818214	100648780	T	1.26E-22	NA	NA	NA	1	1	1	1	1	1
10	rs73262807	35496386	T	2.19E-12	NA	NA	NA	0	0	0	0	0	0
11	rs140909752	10294331	D	9.96E-09	NA	NA	NA	0	0	0	0	0	0
11	rs61881798	16468558	A	3.09E-08	NA	NA	NA	1	1	1	1	1	1
11	rs60562402	47322007	D	1.58E-08	NA	NA	NA	0	0	0	0	0	1
11	rs5792809	78128397	D	1.64E-45	NA	NA	NA	0	0	0	1	1	1
11	rs10899501	78131408	T	6.27E-44	NA	NA	NA	0	0	0	1	1	1
14	rs34813910	60770696	D	2.96E-18	NA	NA	NA	1	1	1	1	1	1
17	rs34872037	79614932	D	1.00E-100	NA	NA	NA	1	1	1	1	1	1

17	rs7405453	79615572	A	1.00E-100	NA	NA	NA	1	1	1	1	1	1

Supplementary Table 7: Brown Hair GWAS. Brown hair vs black GWAS, including analysis following serial conditioning for variants chromosome by chromosome

Chromosome	Locus	Variant	Minor Alle	OR	Z-score	p.value	Annotation	Position	MAF	gene1	gene2	PICS
1	SLC45A1	rs147458259	T	1.39	5.451	5.02E-08	Intergenic	8243102	0.01466	SLC45A1	ERRFI1	1
2	EPHA4	rs7603664	T	1.129	9.734	2.15E-22	Intergenic	222041066	0.424	EPHA4		2
2	EPHA4	rs17349283	G	1.1	7.691	1.46E-14	Intergenic	222089797	0.4534	EPHA4		7
3	ZBTB38	rs35225290	T	1.106	7.788	6.79E-15	Intron	141130835	0.3448	ZBTB38		14
5	SLC45A2	rs16891982	C	0.3529	-39.36	2.25E-308	missense	33951693	0.02281	SLC45A2		1
5	ARL15	rs148906333	T	0.8972	-6.152	7.64E-10	Intergenic	53098206	0.1225	ARL15	NDUFS4	12
5	MAP3K1	rs61055995	T	0.9114	-5.779	7.53E-09	Intergenic	56019064	0.1628	MAP3K1	c5orf67	12
6	IRF4	rs2671427	T	1.146	9.133	8.00E-20	Intergenic	385735	0.3731	IRF4	DUSP22	1
6	IRF4	rs74758148	A	1.326	7.66	1.87E-14	Intergenic	386933	0.04258	IRF4	DUSP22	1
6	IRF4	rs3778607	A	1.14	6.745	1.53E-11	Intron	403799	0.4552	IRF4		1
6	IRF4	rs12211228	C	0.8646	-6.714	1.90E-11	3 prime UTR	408833	0.1419	IRF4		2
6	IRF4	rs4246064	C	0.8505	-9.346	9.10E-21	Intergenic	421196	0.3884	IRF4	EXOC2	3
6	IRF4	rs62389423	A	0.4445	-59.57	2.25E-308	Intergenic	421281	0.172	IRF4	EXOC2	1
6	IRF4	rs143615986	A	0.5531	-17.48	1.91E-66	Intergenic	433066	0.02282	IRF4	EXOC2	1
6	EXOC2	rs974455	G	1.172	9.607	7.50E-22	downstream	485078	0.227	EXOC2		1
6	EXOC2	rs13192740	C	1.09	6.074	1.25E-09	Intron	514991	0.3962	EXOC2		7
6	AKAP12	rs9397040	A	1.079	6.061	1.35E-09	Intron	151580765	0.4175	AKAP12		5
6	EZR	rs923198	C	0.9148	-7.246	4.31E-13	Intergenic	159246717	0.4863	EZR	c6orf99	24
7	LINC-PINT	rs58270997	T	0.9117	-6.695	2.15E-11	Intron	130729394	0.2464	LINC-PINT		3
8	SNX16	rs1452000	G	1.11	7.41	1.27E-13	Intergenic	82767398	0.2634	SNX16		62
9	C9orf66	rs573246	G	0.9319	-5.776	7.67E-09	Intergenic	204014	0.4413	C9orf66	CBWD1	16
9	BNC2	rs7865762	T	0.9162	-5.481	4.23E-08	Intron	16805332	0.1686	BNC2		2
9	BNC2	rs12350739	G	0.8967	-8.818	1.17E-18	Intergenic	16885017	0.3871	BNC2	CNTLN	13
10	ZMIZ1	rs703978	C	0.9263	-6.288	3.21E-10	Intron	80944147	0.4173	ZMIZ1		9
11	PPFIBP2	rs4078279	C	0.9344	-5.504	3.72E-08	Intron	7541511	0.4139	PPFIBP2		11
11	TPCN2	rs72928978	A	1.391	14.55	5.64E-48	missense	68831364	0.1076	TPCN2		3
11	TPCN2	rs1060435	G	1.164	11.91	1.06E-32	utr 3'	68855595	0.4083	TPCN2		2
11	GAB2	rs2292572	T	0.8887	-7.406	1.30E-13	Intron	78052864	0.1587	GAB2		4
11	TYR	rs1042602	A	1.15	10.86	1.80E-27	missense	88911696	0.3674	TYR		1
11	TYR	rs1393350	A	1.165	10.11	5.10E-24	Intron	89011046	0.289	TYR		1
12	CCND2	rs3764032	C	0.8514	-5.989	2.12E-09	Intergenic	4317563	0.04596	CCND2	PARP11	3

12	TMTC3	rs7977437	A	0.833	-11.44	2.68E-30	Intergenic	88708489	0.1456	TMTC3	KITLG	45
12	KITLG	rs12821256	C	1.446	16.7	1.23E-62	Intergenic	89328335	0.1195	KITLG	DUSP6	1
13	SLAIN1	rs765377	T	0.8614	-12.22	2.46E-34	Intergenic	78377834	0.3925	SLAIN1	EDNBR	33
14	SLC24A4	rs12883151	G	1.355	24.09	2.99E-128	intron	92790077	0.4553	SLC24A4		5
15	OCA2	rs1448489	C	1.205	6.703	2.04E-11	Intron	28179879	0.05553	OCA2		3
15	OCA2	rs7169225	G	1.147	5.904	3.55E-09	Intron	28189004	0.2731	OCA2		27
15	OCA2	rs121918166	T	3.255	11.71	1.13E-31	missense	28230247	0.009077	OCA2		1
15	OCA2	rs1800407	T	1.296	11.45	2.33E-30	missense	28230318	0.08559	OCA2		1
15	OCA2	rs4778224	A	0.8856	-5.538	3.05E-08	Intron	28241020	0.1614	OCA2		20
15	HERC2	rs12913832	A	0.4486	-62.16	2.25E-308	Intron	28365618	0.2143	HERC2		1
15	IL16	rs28648707	A	1.109	6.641	3.12E-11	Intron	81534034	0.2068	IL16		58
16	CDK10	rs116927526	T	2.353	5.634	1.76E-08	Intergenic	89743627	0.004391	CDK10	SPATA33	1
16	TCF25	rs9939914	C	1.137	8.71	3.05E-18	upstream	89939929	0.2504	TCF25		1
16	TCF25	rs117243052	T	1.574	6.808	9.88E-12	Intron	89956706	0.01225	TCF25		37
16	MC1R	rs1805005	T	1.324	14.29	2.59E-46	missense	89985844	0.1222	MC1R		1
16	MC1R	rs1805006	A	1.686	8.082	6.36E-16	missense	89985918	0.01312	MC1R		1
16	MC1R	rs11547464	A	1.695	5.799	6.68E-09	missense	89986091	0.00716	MC1R		1
16	MC1R	rs1805007	A	1.822	21.37	2.35E-101	missense	89986117	0.1048	MC1R		1
16	MC1R	rs1805008	T	1.664	18.54	1.03E-76	missense	89986144	0.08953	MC1R		2
16	DEF8	rs117204628	T	1.56	10.94	7.62E-28	3prime UTR	90032455	0.0337	DEF8		1
17	KRT31	rs117612447	T	0.8215	-6.055	1.41E-09		39551099	0.02958	KRT31		1
17	SP6	rs72833466	A	1.169	11.06	2.00E-28	Intron	45948952	0.2769	SP6		3
17	TSPAN10	rs62075722	A	1.261	17.67	7.52E-70	Intron	79611271	0.3571	TSPAN10		11
20	PIGU	rs2424995	A	1.515	17.12	1.02E-65	Intron	33164515	0.09844	PIGU		2
20	ZNF831	rs259960	A	1.089	7.058	1.69E-12	Intron	57826162	0.4838	ZNF831		10

Supplementary Table 8. SNP Heritability of Hair colour. SNP heritability of red, blonde and brown hair, number of SBPs included in each model, the residual heritability following removal of chromosome 16 significant SNPs (red hair only), and removal of all significant SNPS and removal of all significant SNPs and all within 100kb of significant peaks. The final column is the percentage of the full model explained by the SNPs removed.

**Full Model**	**Full model-SNP heritability (h^2)**	**Full model-SNP included**	**Residual model**	**Residual model – SNP heritability (h^2)**	**Residual model – SNP included**	**% of Full Model SNP-h2 explained by genetic variaton not included in residual model**
Red hair – all SNPS	0.403±0.281	8,580,268	Red hair no chr16 significant	0.108±0.0604	8,574,982	73%
			Red hair no significant	0.0499±0.0096	8,571,552	89%
			Red hair no significant +100 Kb	0.0414±0.0076	8,545,261	90%
Blonde hair – all SNPS	0.301±0.061	8,742,765	Blonde hair no significant	0.1132±0.0107	8,720,346	64%
			Blonde hair no significant + 100 SNP	0.0857±0.0068	8,527,341	73%
Brown hair – all SNPS	0.234±0.069	8,554,444	Brown hair no significant	0.1419±0.0204	8,548,233	39%
			Brown hair no significant + 100 Kb	0.1229±0.0152	8,496,741	47%

Supplementary Table 9: eQTL data from GTex of skin gene expression

Locus is the location of the variant, gene_name is the affected gene.

nsnps is the number of tested snps and PPH4 is the probability of colocalisation according to coloc package. Red hair associated variants in sun-exposed skin

Chromosome	Locus	Variant	gene	nsnps	PPH4
6	PKHD1	rs9463733	PKHD1	338	0.918418
16	MC1R	rs1805008	CPNE7	9	0.969414
16	FANCA/SPIRE2	rs3435772	CDK10	125	0.949995
16	MC1R	rs1805008	CDK10	125	0.949995
16	MC1R	rs1805005	CDK10	125	0.949995
16	TUBB3	rs2302898	CDK10	125	0.949995
16	MC1R	rs3212350	CDK10	125	0.949995
16	TCF25	rs4785736	CDK10	125	0.949995
16	MC1R/TCF25	rs8045560	CDK10	125	0.949995
16	FANCA/SPIRE2	rs3435772	FANCA	6	0.960898
16	TCF25	rs7247700	MC1R	30	0.99797
16	TCF25	rs1186238	MC1R	30	0.99797
16	FANCA/SPIRE2	rs3435772	DBNDD1	132	0.993293
16	MC1R	rs1805007	DBNDD1	132	0.993293
17	TSPAN10	rs9747347	TSPAN10	26	0.987846
20	RALY	rs6059655	ASIP	242	0.999745

Supplementary Table 10: eQTL data from GTex of skin gene expression

Locus is the location of the variant, gene_name is the affected gene.

nsnps is the number of tested snps and PPH4 is the probability of colocalisation according to coloc package. Red hair associated variants in not sun-exposed skin

Chromoso	Locus	Variant	gene	nsnps	PPH4
16	MC1R	rs1805008	CPNE7	2	0.960246
16	FANCA/SPIRE2	rs3435772	CDK10	99	0.947822
16	MC1R	rs1805007	CDK10	99	0.947822
16	MC1R	rs1805008	CDK10	99	0.947822
16	FANCA/SPIRE2	rs3435772	ZNF276	1	0.948553
16	MC1R	rs1805007	FANCA	1	0.805048
16	MC1R	rs3212350	TUBB3	11	0.986187
16	MC1R/TCF25	rs8045560	TUBB3	11	0.986187
16	MC1R	rs1805005	GAS8	3	0.932241
16	FANCA/SPIRE2	rs3435772	DBNDD1	117	0.993873
16	MC1R	rs1805007	DBNDD1	117	0.993873
17	TSPAN10	rs9747347	TSPAN10	106	0.935342
20	RALY	rs6059655	ASIP	45	0.993929

Supplementary Table 11: eQTL data from GTex of skin gene expression

Locus is the location of the variant, gene_name is the affected gene.
nsnps is the number of tested snps and PPH4 is the probability of colocalisation according to coloc R package. Blonde hair associated variants in sun-exposed skin

Chromoson	Locus	Variant	gene	nsnps	PPH4
1	PIGV	rs1125358	AL512408.1	202	0.85526
1	PIGV	rs1125358	PIGV	204	0.883802
1	PTAFR	rs1629168	AL353622.1	179	0.958056
1	WDR63	rs1203442	MCOLN2	261	0.892253
2	EN1	rs1303532	LINC01956	24	0.971479
2	MYO1B	rs1261484	STAT4	215	0.961845
3	DIRC2	rs9847240	DIRC2	110	0.967006
3	ZBTB38	rs4683605	ZBTB38	14	0.9801
6	EZR	rs3212308	EZR	82	0.973734
7	SLC12A9	rs1253562	SRRT	47	0.977683
7	SLC12A9	rs1253562	UFSP1	79	0.904186
11	MYRF	rs6189614	TMEM258	55	0.85005
11	MYRF	rs6189614	FADS2	62	0.993674
11	TPCN2	rs3829241	TPCN2	133	0.998465
11	GAB2	rs2292572	GAB2	546	0.89941
16	MC1R	rs1805005	CDK10	507	0.998415
16	MC1R	rs1805008	CDK10	507	0.998415
16	TCF25	rs9939914	SPIRE2	785	0.990521
16	SPATA2L	rs3543245	SPIRE2	785	0.990521
17	WIPF2	rs6206525	MSL1	49	0.957105
17	PDE6G	rs3576341	TSPAN10	69	0.957102
20	RALY	rs6059655	ASIP	249	0.996453
21	SIK1	rs672948	AP001046.1	29	0.952058

Supplementary Table 12: eQTL data from GTex of skin gene expression

Locus is the location of the variant, gene_name is the affected gene.

nsnps is the number of tested snps and PPH4 is the probability of colocalisation according to coloc package. Blonde hair associated variants in not sun-exposed skin

Chromosome	Locus	Variant	gene	nsnps	PPH4
1	PIGV	rs1125358	AL512408.1	154	0.858054
1	PIGV	rs1125358	PIGV	149	0.846986
1	PTAFR	rs1629168	AL353622.1	172	0.956027
1	WDR63	rs1203442	MCOLN2	223	0.864201
1	WDR63	rs1203442	MCOLN3	122	0.898024
2	MYO1B	rs1261484	STAT4	221	0.966367
3	ZBTB38	rs4683605	ZBTB38	6	0.978199
7	SLC12A9	rs1253562	SRRT	60	0.983444
7	SLC12A9	rs1253562	UFSP1	92	0.843271
11	TPCN2	rs3829241	TPCN2	130	0.992484
11	GAB2	rs2292572	GAB2	290	0.854768
16	MC1R	rs1805007	FANCA	2	0.804713
16	MC1R	rs1805005	GAS8	18	0.907267
16	MC1R	rs1805007	DBNDD1	400	0.899756
16	DBNDD1	rs7773340	DBNDD1	400	0.899756
17	WIPF2	rs6206525	MSL1	44	0.965564
20	RALY	rs6059655	ASIP	51	0.987487
21	SIK1	rs672948	AP001046.1	18	0.947979

Supplementary Table 13: eQTL data from GTex of skin gene expression

Locus is the location of the variant, gene_name is the affected gene.

nsnps is the number of tested snps and PPH4 is the probability of colocalisation according to coloc package. Brown hair associated variants in sun-exposed skin

Chromoso	Locus	Variant	gene	nsnps	PPH4
6	EZR	rs923198	EZR	82	0.964396
11	PPIFBP2	rs4078279	AC107884.	54	0.967782
11	TPCN2	rs1060435	TPCN2	133	0.996911
11	GAB2	rs2292572	GAB2	546	0.916813
16	TCF25	rs9939914	SPIRE2	784	0.998233
17	TSPAN10	rs6207572	TSPAN10	69	0.959678
20	PIGU	rs2424995	ASIP	248	0.98229

Supplementary Table 14: eQTL data from GTex of skin gene expression

Locus is the location of the variant, gene_name is the affected gene.
nsnps is the number of tested snps and PPH4 is the probability of colocalisation according to coloc package. Brown hair associated variants in not sun-exposed skin

Chromoso	Locus	Variant	gene	nsnps	PPH4
11	PPIFBP2	rs4078279	AC107884.	72	0.976964
11	TPCN2	rs1060435	TPCN2	130	0.99792
11	GAB2	rs2292572	GAB2	290	0.813979
16	MC1R	rs1805005	GAS8	18	0.849163
20	PIGU	rs2424995	ASIP	51	0.986596

Supplementary Table 15: eQTL data from TwinsUK Multiple Tissue Human Expression Resource of skin gene expression. Locus is the location of the variant, gene_name is the affected gene. nsnps is the number of snps tested and PPH4 is the probability of colocalisation according to coloc R package

Red Hair associated variants

Chromosome	Locus	Variant	gene_name	nsnps	PPH4
16	MC1R	rs1805007	DBNDD1	2	0.998933

Supplementary Table 16: eQTL data from TwinsUK Multiple Tissue Human Expression Resource of skin gene expression. Locus is the location of the variant, gene_name is the affected gene. nsnps is the number of snps tested and PPH4 is the probability of colocalisation according to coloc R package

Blond Hair associated variants

Chromosome	Locus	Variant	gene_name	nsnps	PPH4
1	WDR63	rs1203442	MCOLN2	75	0.859615
5	SLC45A2	rs13289	AMACR	20	0.933465
16	MC1R	rs1805007	DBNDD1	4	0.9989

Supplementary Table 17: eQTL data from TwinsUK Multiple Tissue Human Expression Resource of skin gene expression. Locus is the location of the variant, gene_name is the affected gene. nsnps is the number of snps tested and PPH4 is the probability of colocalisation according to coloc R package

Brown Hair associated variants

Chromosome	Locus	Variant	gene_name	nsnps	PPH4
16	MC1R	rs1805007	DBNDD1	9	0.996569

Supplementary Table 18:
Human candidate genes from blonde GWAS and mouse orthologues

**human candidate gene**	**mouse orthologue**
ACTBL2	Actbl2
ADAMTS12	Adamts12
ADGRV1	Adgrv1
AFG3L1P	Afg3L1
AHNAK	Ahnak
AKAP1	Akap1
AKAP12	Akap12
ALX4	Alx4
AP3M2	Ap3m2
AREG	Areg
ARHGAP24	Arhgap24
ARL15	Arl15
ASB1	Asb1
ATP1B3	Atp1B3
ATXN10	Atxn10
ATXN7L1	Atxn7L1
AXIN2	Axin2
BABAM2	Babam2
BCAS1	Bcas1
BMP4	Bmp4
BMP7	Bmp7
BNC1	Bnc1
BNC2	Bnc2
BTC	Btc
C1Orf127	Gm572
C2Orf91	
C4Orf22	1700007G11Rik
c5orf67	
c8orf86	
CCND1	Ccnd1
CCND2	Ccnd2
CD82	Cd82
CDC42BPA	Cdc42bpa
CDK10	Cdk10
CDK14	Cdk14
CDK15	Cdk15
CDKAL1	Cdkal1
CHL1	Chl1
CLIC5	Clic5
CNTLN	Cntln
CPSF2	Cpsf2
CREB5	Creb5
CRYAA	Cryaa
CXXC4	Cxxc4
DBNDD1	Dbndd1
DCT	Dct
DDHD1	Ddhd1

DDIT4	Ddit4
DIRC2	Dirc2
DLX4	Dlx4
DNAJB2	Dnajb2
DOCK8	Dock8
DSTYK	Dstyk
DUSP22	Dusp22
DUSP6	Dusp6
EDN3	Edn3
EDNRB	Ednrb
EIF3E	Eif3e
EN1	En1
EPHA4	Epha4
EPHB4	Ephb4
ERMP1	Ermp1
ERRFI1	Errfi1
ETV1	Etv1
EXOC2	Exoc2
EZR	Ezr
FGF5	Fgf5
FMN2	Fmn2
FOSL2	Fosl2
FOXE1	Foxe1
FOXI3	Foxi3
FRAS1	Fras1
FREM2	Frem2
FZD1	Fzd1
FZD7	Fzd7
GAB2	Gab2
GABRG3	Gabrg3
GRM5	Grm5
HDAC4	Hdac4
HECTD4	Hectd4
HERC2	Herc2
HNRNPA3	Hnrnpa3
HOXC13	Hoxc13
IL6	Il6
INSC	Insc
INSIG2	Insig2
IQCM	Iqcm
IRF4	Irf4
JAZF1	Jazf1
KAT6A	Kat6A
KCNH1	Kcnh1
KIAA0930	5031439G07Rik
KIAA2026	9930021J03Rik
KITLG	Kitlg
KLF14	Klf14
KRT33A	Krt33A
KRT7	Krt7

KRT86	Krt86
KRTAP17-1	Krtap17-1
LEF1	Lef1
LGR4	Lgr4
LHX2	Lhx2
LURAP1L	Lurap1L
MAP1LC3B2	Gm6054
MAP3K1	Map3K1
MARK3	Mark3
MBNL1	Mbnl1
MC1R	Mc1R
MED13L	Med13l
MITF	Mitf
MKLN1	Mkln1
MRAS	Mras
MRPL39	Mrpl39
MSH5	Msh5
MSI2	Msi2
MSX2	Msx2
MTERF1	Mterf1
MTX2	Mtx2
MYEOV	
MYO1B	Myo1B
MYRF	Myrf
NDUFS4	Ndufs4
NEK6	Nek6
NFIA	Nfia
NOTUM	Notum
NR3C2	Nr3C2
NSG2	Nsg2
OCA2	Oca2
OVOL1	Ovol1
PADI3	Padi3
PARK7	Park7
PARP11	Parp11
PAX3	Pax3
PDE6G	Pde6G
PEBP4	Pebp4
PEX14	Pex14
PIGV	Pigv
PLB1	Plb1
PLK2	Plk2
PLXNB2	Plxnb2
PPFIBP2	Ppfibp2
PPM1A	Ppm1a
PTAFR	Ptafr
PYCR1	Pycr1
RAD51B	Rad51b
RALY	Raly
RCOR3	Rcor3

RGS9	Rgs9
RPL3	Rpl3
RPL34	Rpl34
RPL6	Rpl6
RSPH3	Rsph3a
RSPO2	Rspo2
RXFP3	Rxfp3
SDF4	Sdf4
SGK1	Sgk1
SH3GL3	Sh3gl3
SIK1	Sik1
SLAIN1	Slain1
SLC12A9	Slc12a9
SLC24A4	Slc24a4
SLC38A1	Slc38a1
SLC38A2	Slc38a2
SLC45A1	Slc45a1
SLC45A2	Slc45a2
SNX16	Snx16
SOX6	Sox6
SP6	Sp6
SPATA2L	Spata2l
SPATA33	Spata33
SPZ1	Spz1
STAT4	Stat4
SUCNR1	Sucnr1
SYNE2	Syne2
SYNGR1	Syngr1
TAC4	Tac4
TACC1	Tacc1
TBCD	Tbcd
TCF25	Tcf25
TET2	Tet2
TFAP2C	Tfap2c
THNSL2	Thnsl2
TMEM163	Tmem163
TMEM38B	Tmem38b
TMEM91	Tmem91
TMTC3	Tmtc3
TNFRSF4	Tnfrsf4
TNFRSF9	Tnfrsf9
TPCN2	Tpcn2
TWIST2	Twist2
TYR	Tyr
TYRP1	Tyrp1
UQCRHl	Uqcrh
UTS2	Uts2
VGLL4	Vgll4
WDR63	Wdr63
WIPF2	Wipf2

WNT7B	Wnt7b
XPA	Xpa
ZBTB38	Zbtb38
ZFP36L1	Zfp36l1
ZFYVE16	Zfyve16
ZMIZ1	Zmiz1
ZNF462	Zfp462
ZNF831	Zfp831

Supplementary Table 19

Mouse orthologues of blonde candidate genes expressed in skin or skin appendages

skin expressed mouse genes

Ahnak
Akap1
Alx4
Areg
Arhgap24
Atp1b3
Axin2
Bmp4
Bmp7
Bnc1
Bnc2
Btc
Ccnd1
Ccnd2
Cdk14
Cryaa
Cxxc4
Dct
Ddhd1
Dstyk
Dusp22
Dusp6
Ednrb
En1
Epha4
Ephb4
Ermp1
Ezr
Fgf5
Fosl2
Foxi3
Fras1
Frem2
Fzd1
Fzd7
Hdac4
Hoxc13
Jazf1
Kcnh1
Kitl
Klf14
Krt7
Krt86
Krtap17-1
Lef1
Lgr4
Lhx2

Map3k1
Mbnl1
Mc1r
Mitf
Mkln1
Mrpl39
Msi2
Msx2
Nek6
Nfia
Notum
Ovol1
Padi3
Pax3
Pex14
Pigv
Plk2
Plxnb2
Ppm1a
Sdf4
Sgk1
Sik1
Slc12a9
Slc38a1
Slc38a2
Slc45a2
Sox6
Sp6
Syne2
Tac4
Tcf25
Tfap2c
Tmtc3
Tpcn2
Twist2
Tyr
Tyrp1
Uqcrh
Vgll4
Wnt7b
Zfp36l1
Zmiz1
