# Supplementary material for: Genome-wide study of hair colour in UK Biobank explains most of the SNP heritability
Source: Nat Commun. 2018 Dec 10;9:5271. doi: 10.1038/s41467-018-07691-z (PMC6288091; doi:10.1038/s41467-018-07691-z)
Supplement: Supplementary file 2 — Peer Review File [file 41467_2018_7691_MOESM2_ESM.pdf]

Reviewer #1 (Remarks to the Author):

The manuscript from Morgan et al. presents a fascinating in-depth look at the genetics of hair color in the UK Biobank collection. The authors examine nearly 350,000 individuals of northern European ancestry in which hair color was self-reported to be blond, red, light brown, dark brown, or black. Results are presented for red vs. non-red hair (excluding blond hair), and for blond vs. non-blond hair (excluding red hair), and for brown (light and dark) vs. black (i.e., excluding blond and red) hair. Not surprisingly, hundreds of loci are identified, and the authors supplement their findings with an analysis of eQTL and chromatin mark data.

An important concern has to do with the recent competing manuscript (Hysi et al., published April 2018, and, remarkably, submitted December 2016), in which almost the same dataset was used, and presented as a meta-analysis together with an additional dataset from 23andMe. I was not aware of the Hysi et al. work until now.

In the current work, the authors emphasize findings that were not included in the Hysi et al. manuscript, including a careful analysis of MC1R and red hair: many apparently causal alleles are non-coding, and a penetrance matrix for ~20 different MC1R alleles. They also highlight an association close to POMC for red hair, epistasis between MC1R and ASIP for red hair, and a polygenic risk score for blond hair.

Although the datasets are largely overlapping, I support publication of the current work in that it presents a more biologically informed analysis, and includes some intriguing observations relevant to other complex visible traits. I have a number of comments regarding analysis and presentation that I think will make the current work stronger and more appealing.

1) I find it counterintuitive to present the analysis of MC1R penetrance before the GWAS itself, and I suggest that the authors reorganize the presentation accordingly.

2) In a situation where similar datasets are analyzed, it is important to present what aspects are confirmed, and highlight any discrepancies. Important hallmarks of the Hysi et al. work are the number and identity of the associated loci, and the heritability estimated for red, blond, and black hair. I think the authors of the current work should state whether their analysis revealed any differences from that of Hysi et al.; I also think the authors of the current work should take the opportunity to analyze heritability, or, at a minimum, comment on the estimates (and approach, which seems a little confusing when it comes to population prevalence) used in Hysi et al.

3) It is not clear to me why the authors exclude blond hair from the red hair GWAS, and blond and red hair from the brown hair GWAS.

4) The detailed analysis of MC1R and red hair is interesting, but I find it somewhat confusing that MC1R also turns up in the blond hair and brown hair GWAS tables. Is this just noise due to self-reported classifications or is there a real biological role for MC1R variation in blond and/or brown hair? Perhaps some insight into this question could come from a comparison of the MC1R allelic spectrum in red vs. blond vs. brown hair.

5) I suspect that the signals for POMC and the epistasis signals are probably pretty weak, and I would like to see both the magnitude and the direction of these effects presented more explicitly. From what I can tell, the test for epistasis is a typical quantitative genetic approach, but for the results to be of broad interest, they should be presented and interpreted in the context of prior biologic knowledge, i.e. the known ligand-receptor relationships and previous studies on mouse coat color.

6) I have an analogous concern for MC1R and HERC2/OCA2.

Greg Barsh

Reviewer #2 (Remarks to the Author):

The manuscript, “The genetic architecture of hair colour in the UK population”, by Ian Jackson and colleagues presents an analysis of genetic mapping studies using the persons of European-descent from UK Biobank database. A total sample of 342,234 unrelated persons of white British ancestry” were analyzed using a genome-wide panel of genetic markers. The authors structure their association tests to cover a range of comparisons starting with red vs. brown or black hair color. The authors identify many loci at or near the MC1R gene, a number of which are new amino acid changing mutations and several are new non-coding variations that presumably affect the regulation of gene expression at MC1R. They also identify seven loci that are not near MC1R. Both ASIP, OCA2, and PKHD1, genes encoding ligands for the MC1R receptor were found to interact with MC1R variants demonstrating epistasis and hypostasis. The authors also map blonde vs brown and black hair color and brown vs. black hair color finding many previously uncovered genes including 28 that affect both blonde and black hair. The authors also perform eQT and regulatory enrichment analyses which yield results that are consistent with the proposed actions of pigmentation genes. One very interesting new finding is the importance of recently discovered hair form genes (bot from human GWAS and mouse model databases) in hair color.
Given the recent paper in Nature Genetics by Hysi and colleagues (cited by these authors) which showed an important effect of sex on hair color, I was surprised that these authors didn’t address the importance (or lack of importance) of sex as a factor in hair color variation. Sex is not mentioned a single time in this manuscript.
Referring to the sample of the UK Biobank employed by these researchers as the “UK population” is a bit awkward and implies the others (UK residents with other European and non-European ancestries) who were properly excluded from this analysis are not members of the “UK population”. Although I doubt very much that these authors are making any sort of political statement, I would find some other way to refer to the persons retained in their analyses, since others reporting on or reading this work may misunderstand or misinterpret the naming of the sample as demonstrating or supporting some racialized genetic definition of “UK population”.
Minor comments:
Line 194 – extra period-like symbol at the end of the line
Line 239- probably I wouldn’t use “Curiously” and if I did, or other word like “interestgingly” I would place the commas differently.
Line 251- “genetics association” ◊ “genetic association”
Line 283 and 289 for example. I find leaving off the Oxford comma and little distracting and potentially imprecise. These authors are at least consistent and the journal’s style will prevail regardless.

Reviewer #3 (Remarks to the Author):

The authors mapped the genes associated with hair color using 343,234 samples from the UK Biobank. The significance and novelty of this manuscript is affected by a recent hair color GWAS paper by Hysi et al. (133,238 samples form the UK Biobank and 157,653 samples from 23andme). This manuscript has indeed made a few points that have not been covered by the Hysi et al. paper: 1) epistasis between MC1R and other loci contributing to red hair; 2) genetic continuum from black through dark and light brown to blonde, while red is more like a Mendelian trait; 3) associated genes involving in hair growth or texture.

For the above three points, number 2 is unsurprising, as it has been well known that red hair – largely affected by MC1R – is more like a Mendelian traits with a different genetic model from the

other colors. Number 3 is supported by enrichment analysis, which can only be seen as weak evidence. Number 1 is indeed adding some new knowledge in hair color genetics, but the significance of the finding of epistasis between MC1R and other loci in red hair may not be under wide interest (epistasis in pigmentation genes in general has been well reported before). I therefore think this manuscript is more suitable for a journal of the specific field (e.g. Am J Hum Genet or PLoS Genet).

Reviewer #4 (Remarks to the Author):

The genes involved in human hair colour have been discovered and studied using a variety of techniques. This paper has taken a GWAS approach using the self-reported hair colour data from the UK Biobank of around 350,000 individuals and direct genotyping at 800,000 SNPs and indels. An additional 40 million SNPs were able to be imputed. This has allowed identification of 343,234 of these participants as non-related at the level of 3rd degree relatives. Using this impressive data set the authors were then able to assign genome wide significant associations between SNPs/indels and hair colour using a 5-point scale. The phenotype data is only as good as the self report, never the less this represents an extremely powerful approach to discover the genes and genetic interactions underlying red, blonde, light brown, dark brown and black hair colours. The overall conclusions reached are valid, with more discussion given over for red hair as a special case being driven largely by the MC1R locus. The idea that the polymorphism in genes influencing non-red hair colours, from blonde to black, are on a continuum is probably not that surprising given that non-red hair is based on the continuum of eumelanin content (see Ito and Wakamatsu, J Eur Acad Dermatol Venereol. 2011 Dec;25(12):1369-80, Figure 4), and possible degradation of eumelanin (dilution) that may occur with lighter hair colour. That the genes involved include many that influence hair growth or texture is interesting and emphases that hair colour is not determined only by melanin contributed by the melanocytes, but also a key contribution of keratinocytes to give structural aspects to hair colour.

Specific points:

1. Page 3, In the Introduction the statement that "... ASIP locus and red hair in humans, but the nature of the association has not been explored (8)." Actually, the extended haplotype around the ASIP locus has been equated to a little r allele penetrance for red hair viz.
Duffy et al.. Multiple pigmentation gene polymorphisms account for a substantial proportion of risk of cutaneous malignant melanoma. JID . 2010; 130: 520-8.
"For red hair color, this was markedly so, with the rs4911442*C allele being roughly equivalent to an ''r'' MC1R haplotype."

2. Page 4 " ...(13). This report however, did not distinguish between genes affecting different hair colours". The paper by Hysi analysed hair colour as a single variable from light to dark but did include some red vs black analysis. This sentence should be qualified or removed.

3. Page 5, the distribution of hair colours in the UK Biobank is given as 4.6% red, 11.5% blonde, 41.2% light brown, 37.3% dark brown, and 4.2% black. How does this distribution compare to other published studies in different populations?

4. Page 5, The description of the MC1R alleles and their effects in the section Red hair colour and MC1R is not clear viz.

How many alleles are you discussing before you begin to say "two additional coding variants ... and a variant, rs3212379 ..."

There are many polymorphisms present in the MC1R gene that are ignored, for example see the

supplementary Table 1 from,

García-Borrón et al., MC1R, the cAMP pathway, and the response to solar UV: extending the horizon beyond pigmentation.
Pigment Cell Melanoma Res. 2014 (5):699-720.

In Table 1 there are 16 polymorphisms listed and that should be stated somewhere, together with the population frequency of each allele.
In Table 1 the previously designated highly penetrant "R" D84E and R151C alleles in the homozygous state give frequencies in red hair of 19% and 35% respectively. This is much lower than I would have expected, what is the predominant hair colour of those individuals? Indeed the authors may wish to recheck this Figure and Supplementary Table 1, as I note that the rs1805007 SNP which is R151C, is listed in Figure 1 as R160W, and the rs1805008 SNP which is R160W is listed in Figure 1 as R151C. The errors here should be resolved.
5. There is no designation of MC1R alleles as r or R in the figures, possibly this could be included in another supplementary table giving the odds ratio for red hair for each allele?

6. In Figure 2, what is the locus on chromosome 21 that appears to have a peak and SNP above the genome wide significance level?

7. Page 6, I am skeptical of the statement that the strongest signal of association in the region of MC1R rs34357723 does not originate from any observed amino acid changes. It is highly likely that residual associations (such as an r2 for R151C at 0.2 and R160W at 0.12) could contribute to this. The region on chromosome 16 has very complex LD, the signal for red hair is so strong and is not easy to resolve by adjustments. There is the potential for long range regulatory effects but how much of this is needed to explain the effects on red hair colour beyond the coding variants?

8. Page 7, no need for the extra brackets (10, 17,18) (19, 20)

9. In discussing the additional seven red hair associated loci, the penetrance of these SNPs should be discussed i.e. what is the change in frequency of each SNP between red hair and non-red hair groups. This could also be included as an Odds Ratio, see point 5 above. In addition how much of red hair does each locus explain?

10. Page 7, the epistatic interaction between MC1R coding variants and the ASIP eQTL SNP show in if Figure 3 and Figure S2 could be presented more quantitative in Table format e.g. see Duffy et al. 2010.

11. Page 8, "we discover 213 loci associated with blonde hair colour (… Supplementary Table 3 …). There are 213 entries in Table S3 but some of the loci listed are repetitious e.g. OCA2 and MC1R). What is the true unique number of structural loci that are being identified? The authors do put the caveat in the next sentence(s) and come up with a figure that approx. 1/3 of these are single candidate genes. Rather than giving this loose analysis, it would be better to say that these signals can be reduced to x regions, y of which have been previously recognized as pigmentation or hair colour loci and, z have not previously been described. I find the abstract "Blonde hair is associated with over 200 loci …" misleading or at least not quantitative.

12. Page 8, a GWAS is presented for brown hair colour, it can be assumed the light brown and dark brown classifications are combined for this analysis but the reasoning for not doing separate analysis for these two hair colour groups is not given. If they were done separately as light brown vs dark brown plus black, dark brown vs black, they may have picked up some influence of the MC1R variant alleles on light brown as previously described,

Palmer et al., Melanocortin-1 receptor polymorphisms and risk of melanoma: is the association explained solely by pigmentation phenotype? Am J Hum Genet. 2000, 66(1):176-86

Also, there is some potential for misreporting of red vs light brown hair colours.

13. Page 8, the 56 loci for brown hair colour are not unique, see comment 11 above.

14. Page 9, the eQTL analysis is quite brief given the number of supplementary Tables included. Again I am skeptical of the identification of missense variants in MC1R linked to expression. See point 7. This data could be deleted from the manuscript and repurposed for another paper.

Thank you to the four reviewers for their well considered comments. Our responses are in italic and red below each point

RESPONSE TO REVIEWERS' COMMENTS:

Reviewer #1 (Remarks to the Author):

The manuscript from Morgan et al. presents a fascinating in-depth look at the genetics of hair color in the UK Biobank collection. The authors examine nearly 350,000 individuals of northern European ancestry in which hair color was self-reported to be blond, red, light brown, dark brown, or black. Results are presented for red vs. non-red hair (excluding blond hair), and for blond vs. non-blond hair (excluding red hair), and for brown (light and dark) vs. black (i.e., excluding blond and red) hair. Not surprisingly, hundreds of loci are identified, and the authors supplement their findings with an analysis of eQTL and chromatin mark data.

An important concern has to do with the recent competing manuscript (Hysi et al., published April 2018, and, remarkably, submitted December 2016), in which almost the same dataset was used, and presented as a meta-analysis together with an additional dataset from 23andMe. I was not aware of the Hysi et al. work until now.

In the current work, the authors emphasize findings that were not included in the Hysi et al. manuscript, including a careful analysis of MC1R and red hair: many apparently causal alleles are non-coding, and a penetrance matrix for ~20 different MC1R alleles. They also highlight an association close to POMC for red hair, epistasis between MC1R and ASIP for red hair, and a polygenic risk score for blond hair.

Although the datasets are largely overlapping, I support publication of the current work in that it presents a more biologically informed analysis, and includes some intriguing observations relevant to other complex visible traits. I have a number of comments regarding analysis and presentation that I think will make the current work stronger and more appealing

We thank this reviewer for their support and helpful comments which we have incorporated as described below.

1) I find it counterintuitive to present the analysis of MC1R penetrance before the GWAS itself, and I suggest that the authors reorganize the presentation accordingly.

We have reorganised as suggested

2) In a situation where similar datasets are analyzed, it is important to present what aspects are confirmed, and highlight any discrepancies. Important hallmarks of the Hysi et al. work are the number and identity of the associated loci, and the heritability estimated for red, blond, and black hair. I think the authors of the current work should state whether their analysis revealed any differences from that of Hysi et al.; I also think the authors of the current work should take the opportunity to analyze heritability, or, at a minimum, comment on the estimates (and approach, which seems a little confusing when it comes to population prevalence) used in Hysi et al.

As the analysis methods are different, and our QC excludes SNPs analysed by Hysi et al, an exact comparison of significant SNPs is difficult. However, if we take the 137 significant loci reported by Hysi et al, and ask if we have a significant SNP within 100kb, we find 100 (73%) overlap; 36 are unique to Hysi et al. Of the 163 genes we identify, 93 (57%) are also identified by Hysi et al. The differences are probably due to two factors. We analysed the whole UK Biobank cohort whilst Hysi et al only examined a third. They also analysed the 23andMe cohort which will be more diverse than the UK Biobank. We have added a paragraph to the text, and Supplementary Table 6, which shows the analysis.

We have analysed heritability and have added to the text and a new Supplementary Table 8. We find the SNP heritability for the different colours is 0.403, 0.301 and 0.234 for red, brown and blonde respectively. This is lower than estimates in studies involving pedigrees, but in line with Hysi et al. On

the other hand, we find that our SNP associations account for 90%. 73% and 47% of the SNP heritability of red, blonde and brown hair; much higher than most GWAS.

3) It is not clear to me why the authors exclude blond hair from the red hair GWAS, and blond and red hair from the brown hair GWAS.

We excluded blonde hair from the comparison to try and diminish the possibility of reporting error, in particular "strawberry blonde" individuals reporting as blonde rather than red.

4) The detailed analysis of MC1R and red hair is interesting, but I find it somewhat confusing that MC1R also turns up in the blond hair and brown hair GWAS tables. Is this just noise due to self-reported classifications or is there a real biological role for MC1R variation in blond and/or brown hair? Perhaps some insight into this question could come from a comparison of the MC1R allelic spectrum in red vs. blond vs. brown hair.

Indeed, it seems clear that MC1R does play a role in the blonde to black spectrum, as others have reported taking MC1R as a candidate gene (Duffy et al, Human Molecular Genetics (2004) 13, 447). We have analysed the hair colour of combinations of MC1R coding variants and added Supplementary Table 5, and additional text, showing that although almost all red hair individuals have 2 known functional variants of MC1R, these individuals are only 15% of the total subjects with 2 variants. Blonde hair is found in another 15% of those with 2 variants, and reducing to 11.5% with 1 and 8% with none, whilst black hair rises from 2% with 2, through 4.5% with 1 to 6% with none. We know that MC1R variants are partially penetrant and it appears that in absence of pro-red genetic factors, the MC1R variants increase the probability of having blonde versus black hair. We have analysed each combination of MC1R coding alleles for each hair colour and these are summarised in 4 additional Supplementary Figures 3-6.

5) I suspect that the signals for POMC and the epistasis signals are probably pretty weak, and I would like to see both the magnitude and the direction of these effects presented more explicitly. From

what I can tell, the test for epistasis is a typical quantitative genetic approach, but for the results to be of broad interest, they should be presented and interpreted in the context of prior biologic knowledge, i.e. the known ligand-receptor relationships and previous studies on mouse coat color. *We have added text commenting that the variant at POMC decreases the probability of red hair, and adding context that increased expression of POMC could permit increased signalling through weak MC1R variants. We have also added the epistasis data as a Supplementary Table 3, in which we have the odds ratio for each significant interaction with each MC1R variant*

6) I have an analogous concern for MC1R and HERC2/OCA2.

The HERC2/OCA2 variant reduces the likelihood of red hair (OR <1) We have added text explaining that OCA2 is an albinism gene and suggesting that altered expression of OCA2 may alter the effect of reduced signalling through variant MC1R

Greg Barsh

Reviewer #2 (Remarks to the Author):

The manuscript, "The genetic architecture of hair colour in the UK population", by Ian Jackson and colleagues presents an analysis of genetic mapping studies using the persons of European-descent from UK Biobank database. A total sample of 342,234 unrelated persons of white British ancestry" were analyzed using a genome-wide panel of genetic markers. The authors structure their association tests to cover a range of comparisons starting with red vs. brown or black hair color. The authors identify many loci at or near the MC1R gene, a number of which are new amino acid changing mutations and several are new non-coding variations that presumably affect the regulation of gene expression at MC1R. They also identify seven loci that are not near MC1R. Both ASIP, OCA2, and PKHD1, genes encoding ligands for the MC1R receptor were found to interact with MC1R variants demonstrating epistasis and hypostasis. The authors also map blonde vs brown and black hair color and brown vs. black hair color finding many previously uncovered genes including 28 that

affect both blonde and black hair. The authors also perform eQT and regulatory enrichment analyses which yield results that are consistent with the proposed actions of pigmentation genes. One very interesting new finding is the importance of recently discovered hair form genes (bot from human GWAS and mouse model databases) in hair color.

Again, we thank this reviewer for their support and helpful comments, which we have incorporates as below

Given the recent paper in Nature Genetics by Hysi and colleagues (cited by these authors) which showed an important effect of sex on hair color, I was surprised that these authors didn't address the importance (or lack of importance) of sex as a factor in hair color variation. Sex is not mentioned a single time in this manuscript.

We have added Table 1, showing that females are more likely to self-report red and blonde hair compared to males, and added text to indicate this and to refer to our and others previous published work confirming that there is a real difference using colorimetry. We have added 2 supplementary Figures (7 and 8) where we plot male OR versus female OR for all significant SNPS for both red and blonde hair, showing they are strongly correlated.

Referring to the sample of the UK Biobank employed by these researchers as the "UK population" is a bit awkward and implies the others (UK residents with other European and non-European ancestries) who were properly excluded from this analysis are not members of the "UK population". Although I doubt very much that these authors are making any sort of political statement, I would find some other way to refer to the persons retained in their analyses, since others reporting on or reading this work may misunderstand or misinterpret the naming of the sample as demonstrating or supporting some racialized genetic definition of "UK population".

Thank you for the observation. We have amended the Abstract to say "white, British ancestry, participants in UK Biobank"

Minor comments:

Line 194 – extra period-like symbol at the end of the line

OK, corrected

Line 239- probably I wouldn't use "Curiously" and if I did, or other word like "interestgingly" I would place the commas differently.

We have taken out the word curiously and modified the sentence; see response to referee 4, point 14.

Line 251- "genetics association" ◊ "genetic association"

OK, corrected

Line 283 and 289 for example. I find leaving off the Oxford comma and little distracting and potentially imprecise. These authors are at least consistent and the journal's style will prevail regardless.

We identified and removed a large number of Oxford commas; the few that remain are necessary for meaning

Reviewer #3 (Remarks to the Author):

The authors mapped the genes associated with hair color using 343,234 samples from the UK Biobank. The significance and novelty of this manuscript is affected by a recent hair color GWAS paper by Hysi et al. (133,238 samples form the UK Biobank and 157,653 samples from 23andme). This manuscript has indeed made a few points that have not been covered by the Hysi et al. paper: 1) epistasis between MC1R and other loci contributing to red hair; 2) genetic continuum from black through dark and light brown to blonde, while red is more like a Mendelian trait; 3) associated genes involving in hair growth or texture.

For the above three points, number 2 is unsurprising, as it has been well known that red hair – largely affected by MC1R – is more like a Mendelian traits with a different genetic model from the other colors. Number 3 is supported by enrichment analysis, which can only be seen as weak evidence. Number 1 is indeed adding some new knowledge in hair color genetics, but the significance of the finding of epistasis between MC1R and other loci in red hair may not be under wide interest (epistasis in pigmentation genes in general has been well reported before). I therefore think this manuscript is more suitable for a journal of the specific field (e.g. Am J Hum Genet or PLoS Genet).

We naturally disagree. We think we bring out several key points of general interest not previously reported. Whilst epistasis has been described between pigmentation genes we present a more comprehensive and quantitative analysis. Additionally, we believe that our findings have a wider interest with respect to the importance of genetic interactions and modification of penetrance for a trait with a Mendelian architecture. Furthermore, the finding of skin and hair growth/morphology genes affecting hair colour is important and of wide interest. We do not believe the enrichment analysis is weak evidence.

Reviewer #4 (Remarks to the Author):

The genes involved in human hair colour have been discovered and studied using a variety of techniques. This paper has taken a GWAS approach using the self-reported hair colour data from the UK Biobank of around 350,000 individuals and direct genotyping at 800,000 SNPs and indels. An additional 40 million SNPs were able to be imputed. This has allowed identification of 343,234 of these participants as non-related at the level of 3rd degree relatives. Using this impressive data set the authors were then able to assign genome wide significant associations between SNPs/indels and hair colour using a 5-point scale. The phenotype data is only as good as the self report, never the less this represents an extremely powerful approach to discover the genes and genetic interactions underlying red, blonde, light brown, dark brown and black hair colours. The overall conclusions

reached are valid, with more discussion given over for red hair as a special case being driven largely by the MC1R locus. The idea that the polymorphism in genes influencing non-red hair colours, from blonde to black, are on a continuum is probably not that surprising given that non-red hair is based on the continuum of eumelanin content (see Ito and Wakamatsu, J Eur Acad Dermatol Venereol. 2011 Dec;25(12):1369-80, Figure 4), and possible degradation of eumelanin (dilution) that may occur with lighter hair colour. That the genes involved include many that influence hair growth or texture is interesting and emphases that hair colour is not determined only by melanin contributed by the melanocytes, but also a key contribution of keratinocytes to give structural aspects to hair colour.

We thank this reviewer for their positive comments and deal with their helpful comments below.

Specific points:

Page 3, In the Introduction the statement that "… ASIP locus and red hair in humans, but the nature of the association has not been explored (8)." Actually, the extended haplotype around the ASIP locus has been equated to a little r allele penetrance for red hair viz.Duffy et al.. Multiple pigmentation gene polymorphisms account for a substantial proportion of risk of cutaneous malignant melanoma. JID . 2010; 130: 520-8.
"For red hair color, this was markedly so, with the rs4911442*C allele being roughly equivalent to an ''r'' MC1R haplotype."

We intended this sentence to mean that the molecular basis of the clearly identified genetic interaction has not been explained. Identifying the association with an eQTL increasing the level of ASIP transcript can explain the association. We have modified the sentence to say "Previous studies have defined a role for ASIP in red hair in humans, but its molecular basis is largely unknown". We have added the Duffy (2010) JID reference

2. Page 4 " …(13). This report however, did not distinguish between genes affecting different hair

colours". The paper by Hysi analysed hair colour as a single variable from light to dark but did include some red vs black analysis. This sentence should be qualified or removed.

We have qualified the sentence to read "This report however, mostly considered hair colour as a single ordered variable, including red hair. This has the potential to mask associations due to differences in genetic architecture for separate categories of hair colour. "

3. Page 5, the distribution of hair colours in the UK Biobank is given as 4.6% red, 11.5% blonde, 41.2% light brown, 37.3% dark brown, and 4.2% black. How does this distribution compare to other published studies in different populations?

We now comment that this distribution is very similar to the Queensland population included in Hysi et al, but other European populations have lower red and higher black proportions. We also separate the colours by sex, (new Table 1) and further analyse the sex difference as in Response to Reviewer 1.

4. Page 5, The description of the MC1R alleles and their effects in the section Red hair colour and MC1R is not clear viz. How many alleles are you discussing before you begin to say "two additional coding variants … and a variant, rs3212379 …"

We have clarified this paragraph to make clear that in the analysis we have 13 previously described variants plus the three newly associated ones we find here. We also now simply quote the proportion of red hair individuals with 2 or 1 variants including our newly identified alleles. We have also rearranged the text and figure order as suggested by Reviewer 1 so that the GWAS and identification of novel variants comes first.

There are many polymorphisms present in the MC1R gene that are ignored, for example see the supplementary Table 1 from, García-Borrón et al., MC1R, the cAMP pathway, and the response to

solar UV: extending the horizon beyond pigmentation.Pigment Cell Melanoma Res. 2014 (5):699-720.)

The polymorphisms in MC1R that we discuss are only the ones that are either directly genotyped or robustly imputed in the Biobank cohort. The Garcia-Borron review of course includes all the polymorphisms detected in MC1R whether or not there is any genetic data associating the variant with hair colour.

In Table 1 there are 16 polymorphisms listed and that should be stated somewhere, together with the population frequency of each allele.

We have added a Table (Table 2) with the minor allele frequency and odds ratio of each of the 16 polymorphisms

In Table 1 the previously designated highly penetrant “R” D84E and R151C alleles in the homozygous state give frequencies in red hair of 19% and 35% respectively. This is much lower than I would have expected, what is the predominant hair colour of those individuals? Indeed the authors may wish to recheck this Figure and Supplementary Table 1, as I note that the rs1805007 SNP which is R151C, is listed in Figure 1 as R160W, and the rs1805008 SNP which is R160W is listed in Figure 1 as R151C. The errors here should be resolved.

We have corrected the labelling error in Figure 1, and confirmed the rest of the labels are correct.

With the assignments corrected the penetrance for R151C and R160W are similar to Duffy et al. Also as in Duffy et al D84E, although when homozygous has a modest penetrance of 19%, it has a higher penetrance in combination with other “R” alleles.

We do indeed see a wide range of penetrance across the variants. They do fit previous observations; D294H, for instance has the highest penetrance of the missense variants, as fits with functional analysis. The frame-shift and nonsense variants have the highest penetrance of all.

5. There is no designation of MC1R alleles as r or R in the figures, possibly this could be included in another supplementary table giving the odds ratio for red hair for each allele?

The new Table 2 now has the R vs r designation, plus OR.

6. In Figure 2, what is the locus on chromosome 21 that appears to have a peak and SNP above the genome wide significance level?

This is a very marginal peak, but it is close to SIK1, which is also seen in the blonde GWAS, with an OR of 1.063 (Supplementary Table 4). Given the support for involvement by being in the blonde analysis we have added the SIK1 data to Supplementary Table 1.

7. Page 6, I am skeptical of the statement that the strongest signal of association in the region of MC1R rs34357723 does not originate from any observed amino acid changes. It is highly likely that residual associations (such as an r2 for R151C at 0.2 and R160W at 0.12) could contribute to this. The region on chromosome 16 has very complex LD, the signal for red hair is so strong and is not easy to resolve by adjustments. There is the potential for long range regulatory effects but how much of this is needed to explain the effects on red hair colour beyond the coding variants?

We agree, and have added to the manuscript the likelihood that these are likely to be synthetic associations. In support of this, the frequency of 2-variant red hair individuals is the same whether or not they carry the minor allele at rs34357723; i.e. having this variant doesn't mean you don't need one of the coding variants.

8. Page 7, no need for the extra brackets (10, 17,18) (19, 20)

OK. Corrected

9. In discussing the additional seven red hair associated loci, the penetrance of these SNPs should be discussed i.e. what is the change in frequency of each SNP between red hair and non-red hair groups. This could also be included as an Odds Ratio, see point 5 above. In addition how much of red hair does each locus explain?

The odds ratio for each of the additional SNPs is in Supplementary Table 1, the red hair GWAS. We have analysed how much heritability is explained by the loci (new Supplementary Table 8). MC1R accounts for 73% of the SNP heritability of red hair and the remaining variants for another 17%, leaving only 10% unexplained.

10. Page 7, the epistatic interaction between MC1R coding variants and the ASIP eQTL SNP show in if Figure 3 and Figure S2 could be presented more quantitative in Table format e.g. see Duffy et al. 2010.

We have the epistasis data now presented in a Table (Supplementary Table 3) which includes the odds ratios and P-values for the interactions

11. Page 8, "we discover 213 loci associated with blonde hair colour (… Supplementary Table 3 …). There are 213 entries in Table S3 but some of the loci listed are repetitious e.g. OCA2 and MC1R). What is the true unique number of structural loci that are being identified? The authors do put the caveat in the next sentence(s) and come up with a figure that approx. 1/3 of these are single candidate genes. Rather than giving this loose analysis, it would be better to say that these signals can be reduced to x regions, y of which have been previously recognized as pigmentation or hair colour loci and, z have not previously been described. I find the abstract "Blonde hair is associated with over 200 loci …" misleading or at least not quantitative.

We explain in the text that Our GWAS analyses are conditional, so each associated SNP identified is either independent (as for example the missense variants of MC1R*) or multiple variants may be correlated with the same unidentified variant. We did not intend to say that these are different genes (there are 7 missense variants in MC1R included, for example). We have modified the Abstract to say*

the association is with over 200 genetic variants. Althoguh these variants have a different, independent effect, they may be in LD and hence some of the be grouped in LD blocks. The caveat of 1/3 the variants (now modified to say 64 association signals) is that these are not in LD with others and hence are good candidates for causative variants.

We compare our gene list with Hysi et al and quote the extent of overlap.

12. Page 8, a GWAS is presented for brown hair colour, it can be assumed the light brown and dark brown classifications are combined for this analysis but the reasoning for not doing separate analysis for these two hair colour groups is not given. If they were done separately as light brown vs dark brown plus black, dark brown vs black, they may have picked up some influence of the MC1R variant alleles on light brown as previously described,

Palmer et al., Melanocortin-1 receptor polymorphisms and risk of melanoma: is the association explained solely by pigmentation phenotype? Am J Hum Genet. 2000, 66(1):176-86

Also, there is some potential for misreporting of red vs light brown hair colours.

We now have Supplementary Figures 9 and 10 with GWAS for light brown and dark brown, showing light brown is more like blonde and dark brown more like black in terms of significant peaks.

13. Page 8, the 56 loci for brown hair colour are not unique, see comment 11 above.

As we comment above, as this is a conditional analysis the associated variants are independent. Here and throughout to avoid confusion between loci and genes, we now refer to the GWAS signals as variants where appropriate.

14. Page 9, the eQTL analysis is quite brief given the number of supplementary Tables included. Again I am skeptical of the identification of missense variants in MC1R linked to expression. See point 7. This data could be deleted from the manuscript and repurposed for another paper.

We agree that the associations between missense variants in MC1R and eQTLS at some distance are likely due to synthetic associations due to weak LD . We have modified the sentence to reflect this. This section is brief as the eQTL data does not reveal a great deal, apart from the ASIP eQTL. We nevertheless think it is worth keeping the Supplementary Tables as evidence.

Reviewer #1 (Remarks to the Author):

I think the revised manuscript and reply to reviewers thoughtfully and satisfactorily addresses the concerns raised by myself and the other three reviewers.

With regard to impact for a broad audience, I think the major appeal of the work lies largely in observations with regard to heritability, architecture, and penetrance.

Along those lines, I suggest that the authors consider a somewhat different emphasis with regard to the observations described in the abstract. In particular, I suggest removing the sentence in the abstract regarding POMC, ASIP, and epistasis (since variation in MC1R explains most of the heritability), and replacing it with a sentence that summarizes the findings in lines 223-226 and lines 279-287. (I find it more interesting that while most individuals with red hair have 2 MC1R variants, most individuals with 2 MC1R variants have blond hair).

Reviewer #4 (Remarks to the Author):

The revised manuscript has addressed all my criticisms of the original manuscript and is now much improved with the additional analysis included.

Minor points:

Table 2, columns MAF and OR (initial). The number of significant figures presented here for the values in the Table is excessive and unnecessary. It would be possibly be clearer using 3 (second line 0.009786 as 0.010) and 2 (second line 5.2820 as 5.28) significant figures respectively.

Table 3, The p-value is given as zero in three of these rows. Is this correct?

Response to reviewers:

Reviewer #1 (Remarks to the Author):

I think the revised manuscript and reply to reviewers thoughtfully and satisfactorily addresses the concerns raised by myself and the other three reviewers.

With regard to impact for a broad audience, I think the major appeal of the work lies largely in observations with regard to heritability, architecture, and penetrance.

Along those lines, I suggest that the authors consider a somewhat different emphasis with regard to the observations described in the abstract. In particular, I suggest removing the sentence in the abstract regarding POMC, ASIP, and epistasis (since variation in MC1R explains most of the heritability), and replacing it with a sentence that summarizes the findings in lines 223-226 and lines 279-287. (I find it more interesting that while most individuals with red hair have 2 MC1R variants, most individuals with 2 MC1R variants have blond hair).

Thanks to the reviewer for this comment, it is valuable to have an outside eye on the work. We have amended the Abstract as suggested

Reviewer #4 (Remarks to the Author):

The revised manuscript has addressed all my criticisms of the original manuscript and is now much improved with the additional analysis included.

Minor points:

Table 2, columns MAF and OR (initial). The number of significant figures presented here for the values in the Table is excessive and unnecessary. It would be possibly be clearer using 3 (second line 0.009786 as 0.010) and 2 (second line 5.2820 as 5.28) significant figures respectively.

We have amended as suggested

Table 3, The p-value is given as zero in three of these rows. Is this correct?

The p-value was less that 10^{-4}, we have amended to give this Figure rather that zero.
